# SSRIs target prefrontal to raphe circuits during development modulating synaptic connectivity and emotional behavior

**DOI:** 10.1038/s41380-018-0260-9

**Published:** 2018-10-02

**Authors:** M. Soiza-Reilly, F. J. Meye, J. Olusakin, L. Telley, E. Petit, X. Chen, M. Mameli, D. Jabaudon, J.-Y. Sze, P. Gaspar

**Affiliations:** 1 Institut du Fer à Moulin, Paris, France; 2 Inserm, UMR-S 839, Paris, France; 3 Sorbonne Universités, Paris, France; 4 Department of Basic Neurosciences, University of Geneva, Geneva, Switzerland; 5 Department of Molecular Pharmacology, Albert Einstein College of Medicine, Bronx, New York USA

**Keywords:** Neuroscience, Depression

## Abstract

Antidepressants that block the serotonin transporter, (Slc6a4/SERT), selective serotonin reuptake inhibitors (SSRIs) improve mood in adults but have paradoxical long-term effects when administered during perinatal periods, increasing the risk to develop anxiety and depression. The basis for this developmental effect is not known. Here, we show that during an early postnatal period in mice (P0–P10), Slc6a4/SERT is transiently expressed in a subset of layer 5–6 pyramidal neurons of the prefrontal cortex (PFC). PFC-SERT+ neurons establish glutamatergic synapses with subcortical targets, including the serotonin (5-HT) and GABA neurons of the dorsal raphe nucleus (DRN). PFC-to-DRN circuits develop postnatally, coinciding with the period of PFC Slc6a4/SERT expression. Complete or cortex-specific ablation of SERT increases the number of functional PFC glutamate synapses on both 5-HT and GABA neurons in the DRN. This PFC-to-DRN hyperinnervation is replicated by early-life exposure to the SSRI, fluoxetine (from P2 to P14), that also causes anxiety/depressive-like symptoms. We show that pharmacogenetic manipulation of PFC-SERT+ neuron activity bidirectionally modulates these symptoms, suggesting that PFC hypofunctionality has a causal role in these altered responses to stress. Overall, our data identify specific PFC descending circuits that are targets of antidepressant drugs during development. We demonstrate that developmental expression of SERT in this subset of PFC neurons controls synaptic maturation of PFC-to-DRN circuits, and that remodeling of these circuits in early life modulates behavioral responses to stress in adulthood.

## Introduction

Neuronal circuits undergo experience-dependent axon/synaptic refinement during critical developmental periods that are opportunities for adaptation, but are also windows of vulnerability for maladaptive plasticity with long-lasting consequences on brain function [1–3]. Early-life exposure to selective serotonin (5-hydroxytryptamine, 5-HT) reuptake inhibitors (SSRIs) induces adult anxiety/depression-like phenotypes in rodents [4–7] that are opposite to their mood-enhancing properties in adulthood. Similarly, genetic downregulation of the 5-HT transporter gene (Slc6a4/SERT) causes depression-related behaviors that have a developmental origin [8, 9]. In humans, a lesser-expressing form of Slc6a4 has been associated with an increased risk of developing depression in response to early-life stress [10].

The neurobiological bases for developmental effects of SERT loss-of-function and early-life exposure to SSRIs on emotional behavior remain elusive. However, evidence from rodent [6, 11] and human studies point to the prefrontal cortex (PFC) as a likely target. In humans, the short-allele variant of *Slc6a4* has been linked to volumetric reduction of ventromedial areas of the PFC and to changes of PFC functional connectivity [12, 13]. The PFC plays a prominent role in the control of stress-coping responses, and reduced activity of the PFC has been repeatedly noted in depression [14, 15], while deep-brain stimulation in medial PFC cortical areas can alleviate major treatment-resistant depression [16, 17]. The top–down projections from the PFC to the dorsal raphe nucleus (DRN) could play an important role in these antidepressant effects, as PFC-to-DRN circuits control emotional responses to stressors, and optogenetic stimulation of the PFC-to-DRN circuit influences behavioral responses to stressors [16, 18–20].

In the present study, we identify a subset of PFC pyramidal neurons that transiently express SERT (SERT+) [21, 22] during a critical period of early postnatal life, corresponding to the time when SSRIs induce long-lasting impairments in emotional behaviors [6]. We show that these PFC-SERT+ neurons define a subset of pyramidal neurons in layers 5–6 of the PFC with exclusive subcerebral projections. In particular, PFC-SERT+ neurons form a majority of the cortical inputs to the DRN. Transcriptome profiling of these neurons in SERT-KO mice indicate that *Slc6a4/SERT* controls gene networks involved in axon growth and synaptic development at a time when the PFC–subcortical circuits are developing. Accordingly, complete or cortex-specific conditional SERT ablation leads to increased number of functional PFC glutamatergic synapses onto both 5-HT and GABA neurons of the DRN, and this effect is reproduced by postnatal fluoxetine administration. We further show that the depressive- and anxiety-like symptoms induced by early postnatal exposure to SSRIs are modulated by the PFC-SERT+ neurons in adult life. Thus, our results demonstrate that cortical SERT expression is cell-autonomously required to control the maturation of PFC-to-DRN circuits and is engaged in the top–down control of emotional deficits induced by exposure to SSRIs during early postnatal life, resulting in long-lasting effects on mood.

## Materials and methods

### Animals

All experiments performed in mice were in compliance with the standard ethical guidelines (European Community Guidelines and French Agriculture and Forestry Ministry Guidelines for Handling Animals- decree 87849). All the procedures for generation of SERT^fl/fl^ mice were approved by the Albert Einstein College of Medicine Institutional Animal Care and Use Committee.

All mouse lines were group-housed (3–5 per cage), maintained on a C57BL/6 background, and locally bred under standard laboratory conditions (22 ± 1°C, 60% relative humidity, 12–12 h. light–dark cycle, food, and water ad libitum). In all experiments, both genders were equally represented.

The SERT-Cre knock-in mouse line used here, has been previously characterized [23, 24]. Cre-recombinase expression in this line precisely follows the temporal pattern of expression of the SERT gene [23, 24]. Moreover, since nls-Cre is inserted in exon 2 of the SERT gene, it allows to study SERT-KO mice in the same line :mice carrying 1 or 2 Cre alleles are SERT^+/-^ (SERT^Cre/+^) or SERT^-/-^ (SERT^Cre/Cre^), respectively. For anterograde tracing and array tomography experiments, SERT^Cre/+^ or SERT^Cre/Cre^ mice were obtained from crossings between SERT^Cre/Cre^ male and SERT^Cre/+^ female mice. For localization and RNA-sequencing, SERT^Cre/Cre^ males were crossed with either the RCE-loxP or Ai14 TdTomato reporter mouse lines that conditionally expresses EGFP or TdTomato under the control of the Rosa promoter [25, 26]. The descendants of these animals were backcrossed (SERT^Cre/+^:RCE) to obtain knockout, SERT^Cre/Cre^:RCE, and heterozygotes (SERT^Cre/+^:RCE) mice from the same litters. For ontogenetic analysis of cortical projections, the Emx1b^cre^ mouse line [27] was crossed with the Ai14 TdTomato reporter mouse line [26].

To evaluate the role of cortical vs. raphe Slc6a4/SERT invalidation, SERT^fl/fl^ mice were crossed with Emx1b^Cre^ or Pet1^Cre^ mice [28, 29]. Pharmacological experiments were done in wild-type C57BL/6 mice (Janvier Labs, France) and SERT^Cre/+^ mice. Mouse pups were administered fluoxetine hydrochloride (Tocris, UK) (per os. 10 mg/kg/day in a 5% sucrose) or 5% sucrose from P2 to P14 [6]. In clorgyline experiments, SERT^Cre/+^:RCE pups were treated at P5 with three equally spaced doses of clorgyline (30 mg/kg/8 h, s.c.) (Sigma) and processed for immunohistochemistry 3 h after the last injection (P6). Western blot analyses were done on timed litters of C57BL/6 mice (Janvier Labs, France).

### AAV injections in the PFC

Different replication-defective adeno-associated viruses (AAV) were used. The AAV2/1-CAG-LSL-EGFP-bGH and AAV1-CAG.FLEX-TdTomato-WPRE-bGH (Penn Viral Vector Core, University of Pennsylvania, USA) were used for conditional anterograde tracing and array tomography experiments. The rAAV2/1-CAG-hChR2(H134R)-mCherry (Penn Viral Vector Core) was used for electrophysiology and the AAV5-CaMKIIa-hM4D(Gi)-mCherry, AAV5-hSYN-DIO-hM4D(Gi)-mCherry, AAV8-CaMKIIa-hM3D(Gq)-mCherry, and AAV8-hSYN-DIO-hM3D(Gq)-mCherry (Vector Core, University of North Carolina at Chapel Hill, USA; Addgene, USA) for pharmacogenetic manipulation [30]. All titer ranges were 10^12^–10^13^ particles/ml. Viral injections were done on P4–5 pups anesthetized on ice (2–3 min) and kept on a frozen pad during the procedure. Mice were put in prone position on the frozen pad to maintain the skull flat. After opening of the skin, the PFC was targeted using the inferior cerebral vein (~2–3 mm posteriorly) and the superior sagittal sinus (~2–3 mm laterally) as references. Single unilateral or bilateral injections (50–100 nl each) were performed 1.5 mm deep from the skull surface using a pulled glass capillary (30–50 µm tip diameter; Drummond Scientific company) mounted on a hydraulic micromanipulator MO-10 (Narishige, Japan). The skin was sealed using a tissue adhesive glue (Vetbond #1469, 3M, France), and pups returned to their home cages with the mother. Injected mice were either killed 3 weeks after AAV injection (histology and electrophysiology) or kept in standard housing conditions for subsequent behavioral experiments.

### Brain tissue processing

Mice were killed at P2, P7, P14, P21, or P28. All mice were anesthetized with pentobarbital (0.5 mg/g) and either killed for preparation of fresh tissue slices (western blots, RNA seq) or fixed by intracardiac perfusion of 4% paraformaldehyde in 0.1 M phosphate-buffered saline (PBS; pH = 7.4). Brains were quickly removed and post-fixed overnight in the same fixative solution and cryoprotected for 2 days in 30% sucrose containing sodium azide (0.01%; Sigma, France).

### Immunohistochemistry

The 50-µm-thick coronal sections were prepared using a cryo-microtome (Microm Microtech). Serial sections from the whole brain were collected as series of 4 or 3. Tissue sections were used immediately for immunohistochemistry or stored at −20 °C in a cryoprotective solution (30% ethylene glycol and 30% sucrose in 0.1 M phosphate buffer, pH = 7.4). All the washes and antibody incubations were performed in blocking solution containing 2 g/L gelatin (Merck, USA) and 0.25% triton in PBS (PBGT). Primary antibodies were applied for 24–48 h at 4 °C and secondary antibodies for 2 h at room temperature. The following primary antisera were used: anti-5-HT (rabbit from Sigma, S5545, 1/5000; goat from Abcam, 66047, 1/1000), anti-GFP (rabbit, Molecular Probes, A6455, 1/2500; chicken, Aves, GFP-1020, 1/1000), anti-DsRed (rabbit, Clontech, 632496, 1:400), anti-Ctip2 (rat, Abcam, ab18465, 1/500), anti-Cux1 (rabbit, Santa Cruz Biotechnology, sc-13024, 1/1000), anti-Foxp2 (goat, Santa Cruz Biotechnology, sc-21069, 1/500), and anti-cfos (rabbit, Abcam, 190289, 1/1000). All fluorescent secondary antisera were from a donkey (Jackson ImmunoResearch, 1/200) to visualize multiple markers without cross-reactivity. Sections were mounted with Slowfade Gold antifade with DAPI reagent (Molecular Probes) or with the Prolong Gold antifade reagent (Molecular Probes, USA). For DAB-peroxidase revelation, sections were processed as described previously [21]. Briefly, sections were preincubated in 1% H_2_O_2_ in PBGT (1 h), before the primary antibody. Biotinylated goat anti-rabbit antiserum (Vector Laboratories, BA-1000, 1/200) and streptavidin horseradish peroxidase complex (1:400 in PBS; GE Healthcare Life Science, USA) were used as secondaries. Peroxydase revelation was done with 0.02% diaminobenzidine (DAB, Sigma)/ Nickel Ammonium Sulfate (0.6%, Sigma, France) in Tris-HCl (0.05 M, pH 7.6) and H_2_O_2_ (from 0.00015 to 0.0012%; Sigma, France). Sections were mounted on glass slides (Superfrost Ultra Plus, Thermo Scientific), air-dried (24 h), dehydrated, and coverslipped with Eukitt (Sigma, France).

### Image acquisition

DAB-revealed sections were imaged using a slide scanner (Nanozoomer 2.0-HT C9600, Hamamatsu, Japan) objective X20 and analyzed with the NDP View2 software (Hamamatsu, Japan). For illustration purposes, bright-field images were exported in tiff format from the nanozoomer images with NDP viewer or captured with a Cool SNAP camera mounted on a provis microscope (Olympus). For immunocytochemical analyses, fluorescence images were acquired on a Leica DM6000 fluorescence microscope using either 40 × /1.25 N.A Plan-apochromat objective (for cell bodies) or a 63 × /1.4 N.A Plan-apochromat (for axons). Confocal images were acquired on a Leica SP5 confocal microscope equipped with an Argon laser (488 nm excitation), a Diode 561 nm and HeNe 633 nm (at a 1024 × 1024 pixel resolution).

### In situ hybridization

Brains were sectioned in a cryostat (Leica Microsystems, France) as 20-μm-thick sections. SERT cRNA probe [21] (nucleotides 1510–2009) inserted in pBluescript SKII2 (Stratagene, La Jolla, CA) was linearized using BamHI (#FD0054, Fermentas, France) Antisense RNA synthesis was with the T7 polymerase (#EP0111, Fermentas, France). RNA probe (0.1–1 μg/ml) in hybridization buffer (50% formamide, 10% dextran sulfate, 1X Denhardt’s, 5X SSC, and 250 μg/ml transfer RNA) and incubated at 65 °C for 10 min. Hybridization buffer (350 μl) was added to sections and incubated overnight. Sections were washed with PBS Triton X-100 0.1%, incubated with anti-digoxigenin (1:1000, #11093274910, Roche, France) at 4 °C overnight, washed sequentially in PBS and NTMT buffer (Tween 10%; Tris-HCl 1 M, pH 9.5; MgCl_2_ 1 M; NaCl 5 M; H_2_O), and incubated at 37°C with NBT/BCIP (#11383213001/ #11383221001; Roche, France) for revelation (2–24 h). Reaction was arrested in PBS and sections were mounted in mowiol-Dabco (25 mg/ml, Sigma, France).

### Array tomography

After tissue processing, as described above, brains were sliced to 300-μm-thick coronal sections with a vibratome. Thick sections containing either the DRN, the mediodorsal thalamic nuclei (MD), or the basolateral amygdala (BLA) were processed for array tomography as described [31, 32]. Briefly, sections were dehydrated (50 and 70% ethanol, 5 min each step at room temperature) and equilibrated in a mixture (1:3) of 70% ethanol and LRWhite resin (medium grade, Electron Microcopy Sciences, USA; #14380) for 5 min, and then 2 × 5 min in pure LRWhite. After equilibrium in LRWhite overnight at 4°C, sections were flat embedded between a glass slide and a sheet of ACLAR plastic (Electron Microcopy Sciences, USA; #50425-10), and polymerized for 24 h at 55 °C [32]. After embedding, regions of interest were excised from the flat-embedded sections and glued with a superglue to EMBed 812 blocks for ultrasectioning. After adhesive cement diluted in xylene was applied to the top and bottom of the block of tissue, series of at least 25–70-nm-thick serial sections were cut in ribbons using Jumbo Histo Diamond Knife (Diatome, 578, USA) and an ultramicrotome (Leica, France). Ribbons were mounted on glass coverslips precoated with 0.1% gelatin and 0.01% chromium potassium sulfate. After air-drying, coverslips containing the ribbons were placed on a hot plate (60 °C) for 30 min and then stored at room temperature until use.

For immunofluorescence, all antibodies used have been characterized in previous array tomography studies. The following antibodies were used: anti-DsRed (rabbit, 1:400; Clontech, USA; #632496) [33], anti-VGLUT1 (guinea pig, 1:1000; Millipore, USA; AB5905), anti-VGLUT2 (guinea pig, 1:1000; Millipore, USA; AB2251), and anti-glutamate decarboxylase 65 (GAD2) (rabbit, 1:200; Cell Signaling Technology, USA; #5843) [32, 34]. The anti-synapsin 1 (rabbit, 1:400; Cell Signaling Technology, USA; #5297) was used to identify synaptic boutons [34] and the anti-tryptophan hydroxylase antisera (sheep, 1:200; Millipore, USA; AB1541) was used to identify 5-HT neurons [32, 34]. Multiple combinations of these antibodies were applied in a random order and subsequently imaged. To achieve this, after each round of immunolabeling, the antibodies were eluted from the sections, and they were reimmunolabeled with a different set of antibodies and re-imaged. The tryptophan hydroxylase labeling was included in each round of immunolabeling and then used as a reference for sections alignment. For immunolabeling, sections were encircled with a hydrophobic barrier pen (ImmEdge; Vector Labs, USA; #H-4000) and preincubated for 5 min in a blocking solution (0.05% Tween; 0.1% bovine serum albumin in Tris buffer saline; pH 7.6). Subsequently, primary antisera were diluted in the blocking solution and incubated with sections for 2 h.

Sections were rinsed with PBS 3 × 10 min, and appropriate fluorescent-conjugated secondary antisera raised in donkey were used (Alexa 488, Alexa 647, and CY3, 1:200; Jackson ImmunoResearch, USA). Secondary antisera were centrifuged at 15,000*g* for 3 min before use. Sections were incubated with the secondary antisera for 24 min and rinsed as before. The coverslips containing the sections were mounted on glass slides using a glycerol-based mounting solution (90% glycerol in PBS, pH 9) and imaged within the following 3 h. Antibodies were eluted after imaging with 0.02% SDS and 0.2 M NaOH in distilled H_2_O for 20 min. After 2 × 10 min washes in distilled H_2_O, coverslips were air-dried and placed on a hot plate (60 °C) for 30 min. Negative controls omitting primary antisera were run to corroborate the complete elution of primary antibodies.

Serial sections were imaged on a Leica DM6000 fluorescence microscope using a Leica 63X NA 1.4 Plan Apochromat oil objective, a CoolSNAP EZ camera, and Metamorph software (Molecular Devices, USA). Multiple channels of serial images were aligned and converted into stacks with Fiji software and the StackReg and MultiStackReg plugins, using the tryptophan hydroxylase labeling as a reference channel [34]. We analyzed two stacks of at least 25 images per region (i.e., DRN, MD, and BLA) per animal to obtain a mean value of number of synaptic boutons per µm^3^ of tissue for each mouse. The quantitative analysis is typically done using a sampling mask of 90 μm X 90 μm. Images were converted to binary images using the threshold function and in some cases manually adjusted to exclude non-specific background. Axonal boutons were identified by the presence of double-labeled voxels, including VGLUT1, VGLUT2, or GAD2 in addition to synapsin labeling in each case as previously described [34], yielding density values per tissue volume (puncta per μm^3^). To avoid underdetection of adjacent objects located close to one another or in different synaptic compartments (e.g., synaptic boutons and tryptophan hydroxylase labeling), we used the “dilate” function that introduces a mask expansion in the size of one of the objects < 0.2 μm. In these analyses, we used this resulting dataset to quantify the density of labeled overlapping voxels [34]. For the statistical analysis, array tomography data obtained from two stacks per animal were averaged to generate a mean density value per mouse. Data were collected and analyzed, blind to the genotype/treatment.

### Western blot

Raphe tissue was macroscopically dissected on ice using the aqueduct as landmark, from brains of P7, P14, P21, and P28 mice. Tissue from 2−3 brains was pooled, and 600–900 µl of lysis buffer [Tris 100 mM pH 7.6, EDTA 0.5 M pH 8, 1% Triton X-100 and protease inhibitor cocktail (#P8340, Sigma, France)] was added to the tissue and sonicated on ice for 5 mins. Samples were centrifuged at 13,000 *g* (30 min at 4°C), supernatants were collected, and an aliquot from each sample was used for protein determination (bicinchoninic assay). A total of 50 µg of each protein extract sample was subjected to SDS-PAGE, using NuPage 4–12% Bis-Tris polyacrylamide gels (Invitrogen, France). Proteins were blotted onto a 0.45-µm nitrocellulose membrane (Thermo Fisher, France). Membranes were blocked (1 h) in 5% non-fat milk dissolved in PBS (pH = 7.6) −0.1% Tween (Sigma-Aldrich, France) (PBST) and incubated overnight in 1:1000 rabbit anti-VGLUT1 (Synaptic Systems, Germany, Cat. # 135302) or 1:1000 rabbit anti-GAPDH (Bethyl Labs., USA, Cat. # A300-641A-M) at 4°C. After three rinses in PBST, membranes were incubated in Alexa Fluor 790-conjugated donkey anti-rabbit secondary antiserum (1:10000, Jackson ImmunoResearch, USA, #711-655-152) at room temperature for 90 mins. Revelation was done using a Li-Cor-Odyssey infrared Imaging System (Li-Cor, Germany, #ODY-Ø398). Band intensities were analyzed with ImageJ software. Data were expressed as the ratio between the band intensity of VGLUT1 and GAPDH.

### Electrophysiology

Mice were anesthetized (Ketamine/Xylazine; 50 mg/10 mg/Kg i.p.) and brain slices were prepared as previously described [33]. After decapitation, brains were rapidly isolated and placed in carbogenated ice-cold 95% O_2_/5% CO_2_-equilibrated solution containing (in mM): cholineCl (110), glucose (25), NaHCO_3_ (25), MgCl_2_ (7), ascorbic acid (11.6), Na^+^-pyruvate (3.1), KCl (2.5), NaH_2_PO_4_ (1.25), and CaCl_2_ (0.5). Coronal brain slices of 250 µm thickness containing the DRN were prepared and transferred for 10 min to a warmed solution (34 °C) of identical composition. Subsequently, slices were stored in carbogenated artificial cerebrospinal fluid (ACSF) containing (in mM): NaCl (124), NaHCO_3_ (26.2), glucose (11), KCl (2.5), CaCl_2_ (2.5), MgCl_2_ (1.3), and NaH_2_PO_4_ (1) for 50 min prior to recordings. Slices were then transferred to a recording chamber, in which they were immersed in warmed ACSF (30 °C), with a flow rate of 2.5 ml/min. Patch clamp recordings in voltage-clamp mode were made under an Olympus-BX51 microscope (Olympus, France), using borosilicate glass pipettes (2.5–4 MΩ; Phymep, France) filled with an internal solution containing (in mM): CsCl (130), NaCl (4), MgCl_2_ (2); EGTA (1.1), HEPES (5), Na_2_ATP (2), Na^+^-creatine-phosphate (5), Na_3_GTP (0.6), and spermine (0.1). The liquid junction potential was −3 mV. Currents were amplified, filtered at 5 kHz, and digitized at 20 kHz. Series resistance was monitored by a voltage step of −4 mV (0.1 Hz). Experiments were discarded if the access resistance increased more than 20%.

Putative 5-HT neurons were identified based on their large (>30 pF) membrane capacitance and medial position within the DRN [34, 35]. Putative non-5-HT neurons were identified based on their location lateral to the midline within the DRN and their small membrane capacitance ( < 20 pF) [36]. To further validate this approach, in certain cases DRN neurons in brain slices were fluorescently labeled by electroporation via a glass stimulation pipette filled with (in mM): K-Gluconate (140), KCl (5), HEPES (10), EGTA (0.2), MgCl2 (2), Na2ATP (4), Na3GTP (0.3), creatine phosphate (10), and Alexa 488 nm (1). The stimulation pipette was placed on the cellular membrane, after which 40 square −12 V pulses of 1 ms at 50 Hz were delivered. Subsequently, after a few hours of fixation in 4% PFA at 4 °C, brain slices were processed for immunohistochemistry using a rabbit polyclonal antibody against TPH2 (1:2000, Novus Biologicals, NB100-74555) to identify 5-HT neurons. This analysis confirmed the criteria used to identify 5-HT vs. putative GABAergic TPH2-negative neurons (Supplementary Fig. 7).

Optogenetically evoked excitatory postsynaptic currents (oEPSCs) at synapses from the PFC to putative 5-HT and GABA DRN neurons were evoked via light pulses (470 nm, 1–10 ms; at 0.1 Hz) delivered with an LED (CoolLed, UK) illumination system. All recordings were performed in the presence of 100-µM picrotoxin to pharmacologically block GABA-A receptor-dependent synaptic currents. Input–output relationships for AMPA receptor (AMPAR-EPSCs) were determined by voltage-clamping DRN neurons at −50 mV and stimulating PFC inputs at different light intensities (0.1, 1, 2, 3.5, 5.6, 7.2, 8.6, and 9.8 mW). Subsequently ten sweeps of AMPAR-EPSCs in response to these different stimulation intensities were averaged and the peak value was scored. AMPAR/NMDAR ratios of oEPSCs were obtained by taking the peak value of AMPA-EPSCs at −50 mV (average of 10 sweeps), and the late component of the EPSC at + 40 mV, 50 ms after the onset for NMDA-EPSCs (average of ten sweeps).

### Cell-sorting and RNA-sequencing

Transcriptome profiling of PFC SERT+ neurons was performed on P7 SERT^Cre/+^:RCE (*n* = 3) and SERT^Cre/Cre^:RCE (*n* = 3) littermates. The PFC region containing the EGFP-positive neurons was microdissected as follows. Brains were placed in cold aCSF (119 mM CaCl, 2,5 mM KCl, 2 mM MgCl, 2,5 mM CaCl_2_, 1 mM Na_2_HPO_4_, 26,2 mM NaHCO_3_, and 20 mM glucose) and embedded in low-melting agarose 4% (Carl Roth, Switzerland) and cut into 600-μm vibratome sections in cold and oxygenated ACSF with 3 mM kynurenic acid (Sigma-Aldrich, Switzerland). Slices containing EGFP-positive cells were selected under a fluorescent stereomicroscope Leica M165FC and incubated with oxygenated aCSF/kynurenic at RT for 1 h. Slices were then chemically digested for 10 min with 0.5 mg/ml Pronase (Sigma-Aldrich, Switzerland), then washed with aCSF/kynurenic acid for 5 min, ACFS/kynurenic/BSA 10 min, and aCSF/kynurenic 5 min 2 times. The area of interest was dissected under a fluorescence stereomicroscope and mechanically dissociated on ice. Fluorescent cells were sorted on the MoFlo Astrios (Beckman) and collected in aCSF. Reverse transcription and pre-amplification of the cDNAs were achieved using the SMART-Seq v4 Ultra Low input RNA kit for Illumina (Clontech) on intact cells. RNA-sequencing libraries of the harvested cDNA were prepared using the Illumina Nextera XT DNA Sample Preparation kit. Libraries were multiplexed and sequenced as 50 bp pair-end reads using the Illumina HiSEQ2000 platform at an expected depth of 60 M reads. RNA-sequencing was performed in the Genomic Core Facility, Faculty of Medicine, University of Geneva, Switzerland. The obtained RNA-seq data were prepared and analyzed as follows: (1) the sequenced reads were aligned on mouse reference genome assembly (GRCm38), using TopHat [37]. (2) the number of reads per transcript were calculated with HTSeq, a python framework to work with high-throughput sequencing data [38]. The obtained read counts were normalized by the library size using DESeq R package distributed within the Bioconductor project [39]. All the analyses were computed on the UG Vital-It cluster administrated by the Swiss Institute of Bioinformatics (SIB). Top differentially expressed genes are identified by the fold change and statistical significance of the difference in expression levels [−1 < log2 (fold change) > 1; and uncorrected *P*-value < 0.05]. Gene ontology analysis was done using DAVID Bioinformatics Resources 6.8 (Beta), NIAID/NIH [40]. The Fisher’s exact test was used to statistically evaluate enrichment folds of gene ontology terms. As background, the *Mus musculus* (mouse) whole genome was used.

### Behavioral studies

Female and male mice were tested separately for emotional behaviors following the same series of behavioral tests starting at P80. Testing started with the novelty-suppressed feeding test, followed by the 2-day forced swim test (FST) and the locomotor activity measurements with 7 days of interval between each test. All behavioral testing was done during the light cycle between 10 am and 5 pm. Mice were acutely injected 30 mins before the testing session with clozapine N-oxide (CNO, i.p. 1 mg/kg) using 0.9% NaCl as a vehicle.

Novelty-suppressed feeding test (NSF) was done as described before [4] using a plastic box (50 cm × 80 cm × 20 cm) as arena in which the floor was covered with 3-cm of wooden bedding. Twenty-four hours before the test, mice were food deprived in their home cage. Mice were individually weighed to determine % weight loss. During the test session, two food pellets were placed on a circular white filter paper (12 cm diameter) located in the center of the arena. Mice were placed in a corner of the box and the latency to approach the pellet and begin feeding were manually recorded. After the test, the weight of pellet consumption in the home cage during 5 min was registered to control for possible appetite differences.

FST was carried out over two consecutive days as described [41] using a glass cylinder (40 cm × 20 cm diameter) filled with water (23–24 °C). Mice were tested once a day for 6 min. In the first day, mice were tested in a drug-free condition, while in the second day mice were acutely injected with either CNO or SAL 30 mins before the test. All the swim sessions were videotaped and the time of immobility within the tank was quantified post hoc by a blind observer from the videos. After testing, all mice were dried with paper towels and returned to their home cages.

Locomotor activity. Mice were introduced in a circular corridor (4.5 cm width, 17 cm external diameter) equipped with four infrared beams (1.5 cm above the base) equidistantly located every 90° (Imetronic, Pessac, France). Locomotor activity was recorded during 30 min, and interruptions of two successive beams were automatically registered by a computer.

### Statistical analysis

All data were analyzed using SPSS Statistics 20 (IBM, NY, USA). Array tomography, ontogenetic studies, electrophysiology, and behavioral data were analyzed by one-way or multifactorial ANOVA, (detailed in figure legends). RNA-seq data were analyzed by *t* test. Sample size was established by previous pilot and published studies. Animals of each genotype/gender were randomly assigned to different studies. When data did not comply with the homogeneity of variances ANOVA assumption, Welch’s *t* test was performed (Fig. 3d and Supplementary Figure 2c). Data distribution was assumed to be normal, and two-tailed analyses were carried out with a significance level established at *p* < 0.05.

## Results

### Molecular identity of PFC-SERT+ neurons

**Fig. 1:**
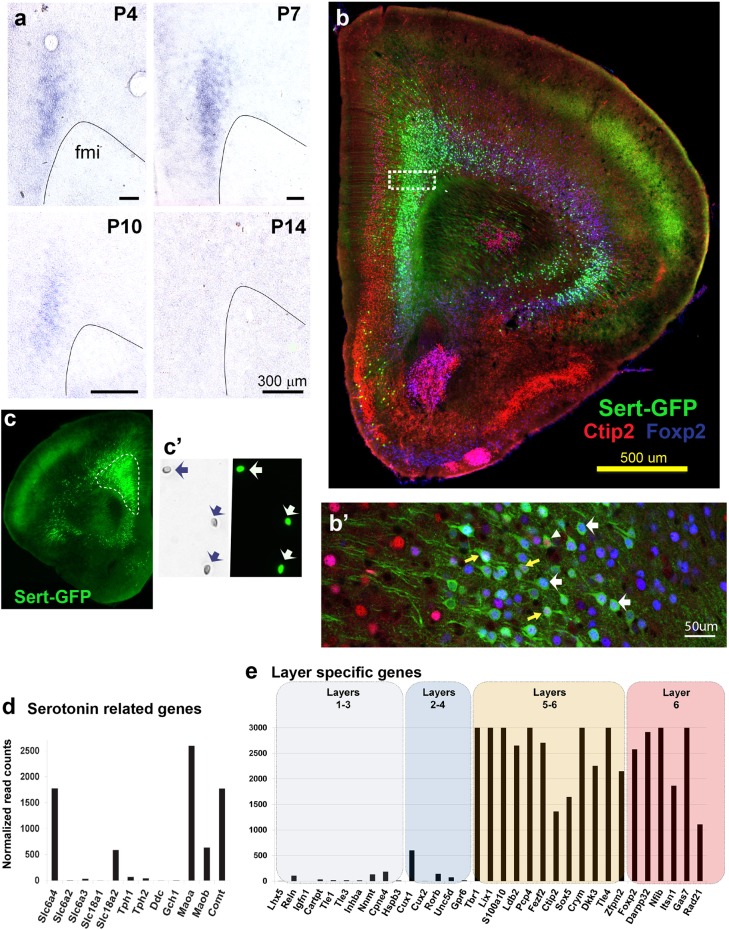
Molecular identity of SERT+ neurons in the prefrontal cortex (PFC). **a** Transient SERT expression in the PFC during postnatal development revealed by in situ hybridization on coronal sections through the frontal pole (postnatal ages (P): 4, 7, 10, and 14). **b** Immunolabeling against Ctip2 (layer 5), Foxp2 (layer 6), and GFP (SERT^Cre/+^) in the PFC. **b’** SERT-GFP neurons often colocalize with Ctip2 (arrowheads), Foxp2 (white arrows), or both (yellow arrows). **c** GFP-expressing neurons from the PFC of SERT^Cre/+^:RCE mice were dissected and subsequently isolated using FACS (in **c'**, individual cells indicated by arrows). **d** Expression levels of monoamine-related transcripts in isolated PFC SERT-GFP neurons from (**c'**) represented by the normalized read counts obtained after deep transcriptome sequencing. **e** Expression levels of cortical layer-specific molecular markers after transcriptome analysis indicating an enrichment of deep layer markers (layers 5 and 6) in PFC SERT-GFP neurons

In the adult mammalian brain, Slc6a4/SERT is mainly expressed by 5-HT-synthesizing raphe neurons [42]. However, during development, Slc6a4/SERT is also transiently expressed in several glutamatergic neurons that show the surprising capacity of re-uptaking 5-HT with high affinity, as shown in different species including humans [21, 22, 43, 44]. In the mouse cerebral cortex, high levels of developmental Slc6a4/SERT expression were found in the PFC and orbital cortical regions and noted to be exclusively present in pyramidal neurons, starting at E18 [23]. To investigate the precise developmental time course of expression of this transcript, we performed an in situ hybridization study that showed postnatal Slc6a4/SERT expression in PFC neurons up to P10 (Fig. 1a). Further characterization using a reporter mouse line (SERT^Cre/+^:RCE-EGFP) confirmed that the majority of the PFC SERT-expressing neurons were located in the deep layers of the prelimbic, infralimbic, and lateral orbital cortical regions, with more scattered expression in the deep layers of the somatomotor areas (Fig. 1b, c). Further, triple immunofluorescent labeling in the SERT^Cre/+^:RCE-EGFP mouse using layer-specific markers such as Ctip2 (for layer 5) or Foxp2 (for layer 6) confirmed that all the PFC-SERT+ neurons are located in these cortical layers (Fig. 1b, b'). Thus, 90% of SERT + neurons express Foxp2 and 70% of SERT+ neurons express Ctip2, among which a large proportion of which coexpressed Foxp2 (Fig. 1b'). Interestingly, the SERT+ neurons represent a subset of the total of layers 5–6 neuron population (39% of the Ctip2 + cells, and 44% of the Foxp2+ cells). These results indicate that an area-restricted subset of layers 5–6 projection neurons expresses Slc6a4/SERT during early postnatal life.

Next, we sought to better understand the molecular identity of PFC-SERT+ neurons, in particular to determine whether they have the classical molecular machinery of monoaminergic neurons, such as the capacity of synthesizing and degrading monoamines. To this end, we performed a transcriptome-wide analysis using deep RNA-sequencing. Gene expression of PFC-SERT+ neurons was analyzed from fluorescence-activated cell sorting (FACS)-isolated SERT^Cre/+^:RCE-EGFP neurons (Fig. 1c, c’, Supplementary Table 1). This was done at P7 when PFC-SERT+ neurons have high levels of Slc6a4/SERT expression (Fig. 1a). As indicated by mRNA expression levels and histochemistry, *Slc6a4/SERT* and *Slc18a2* (vesicular monoamine transporter 2, VMAT2) are the only monoamine transporters present in these neurons (Fig. 1d, Supplementary Figure 2). On the other hand, genes required for 5-HT synthesis such as the enzymes tryptophan hydroxylase (*Tph1*, *Tph2)* or aromatic acid decarboxylase (*Ddc)*, as well as necessary co-enzymes of the BH4 synthesis pathway (*Gch1)* [45] are not expressed (Fig. 1d). In contrast, the PFC-SERT+ neurons express several of the catabolic enzymes for monoamines, *MaoA*, *MaoB*, and *Comt* (Fig. 1d, Supplementary Figure 1, 2). This demonstrates that PFC-SERT+ neurons are not monoaminergic and suggests that a main role of Slc6a4/SERT in these neurons is to remove 5-HT from the extracellular space. Indeed, although no 5-HT immunolabeling was noted in layer 5–6 cortical neurons at P6, 5-HT accumulation became visible after pharmacological inhibition of monoamine oxidase A with the specific inhibitor, clorgyline. This 5-HT staining in the PFC-SERT neurons was abolished upon fluoxetine co-administration (Supplementary Figure 1).

Additionally, regarding the identification of the cortical neuronal subtypes, our transcriptome data showed glutamate but no GABA-related or glial-related genes in the PFC-SERT+ neurons, confirming their classification as pyramidal neurons with an enrichment in expression of molecular markers of layers 5–6 [46] (Fig. 1e), as also shown by our immunohistochemical observations (Fig. 1b’). However, to obtain a finer grain classification of the PFC-SERT+ neuron subtypes would require single-cell RNA-sequencing, as done in previous analyses of cortical pyramidal neurons [47].

### Slc6a4/SERT controls developmental gene networks in PFC-SERT+ neurons

To shed light on the possible functional role of developmental Slc6a4/SERT expression in PFC neurons, we performed a transcriptome profiling analysis of FACS-isolated PFC-SERT+ neurons as described above (Fig. 1c, c’), but in this case comparing gene expression profiles between control heterozygote and SERT-KO mice (SERT^Cre/+^:RCE-EGFP and SERT^Cre/Cre^:RCE-EGFP, respectively) (Supplementary Figure 3a). This analysis identified that a total of 137 genes differentially expressed when *Slc6a4/SERT* are invalidated (Supplementary Table 1). Many of the upregulated or downregulated genes were expressed at moderate-to-high levels in the control condition (Supplementary Figure 3b). Among the 43 genes with significant changes, 30 were downregulated (Supplementary Table 2), while only 13 were upregulated (Supplementary Table 3). Many of these genes are involved in cytoskeleton interactions (*Stmn1-rs1, Plcg1, Tuba1c,* and *Antxr1*), neurite/axon outgrowth (*Pcdhgc4, Gna14, Pcdha12, Pcdha3, Clstn1, Clstn3, Jun, Limk1, Cdh18, Unk, Nedd9,* and *Plp1*), and synapse development (*Pcdhgc4, Gna14, Telo2, Pcdha12, Pcdha3, Pla2g6, Sh3bp5l,* and *Sncaip*). Further examination of the impact of *Slc6a4/SERT* invalidation on gene networks using gene ontology analysis revealed that the axon/synapse maturation/function, growth regulation, and signaling-related developmental process networks were the most affected (Supplementary Figure 3c and Supplementary Table 4).

Overall, these findings support a role of PFC *Slc6a4/SERT* expression in the maturation of PFC-SERT+ neuron-projections and circuit assembly.

### PFC-SERT+ neurons contribute to corticothalamic and subcerebral pathways

**Fig. 2:**
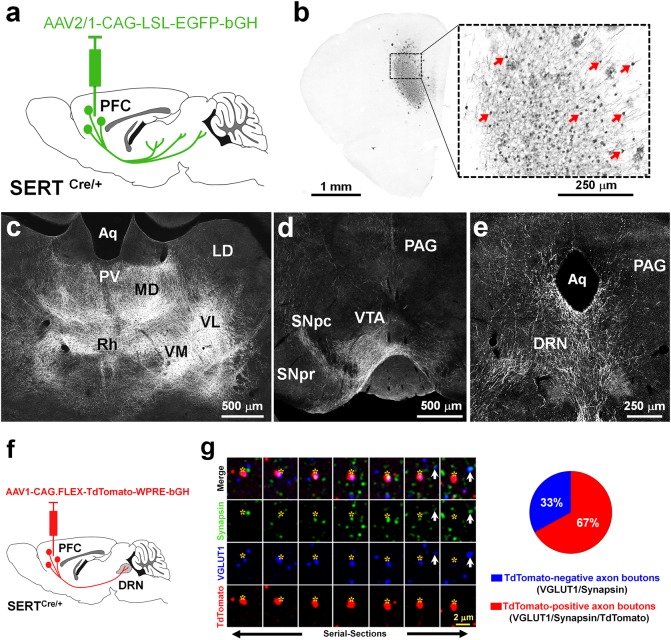
Subcortical brain targets of PFC-SERT+ neurons and their large contribution to the PFC-to-DRN synaptic circuit. **a** Schematic illustration of the injection site in the PFC. The AAV2/1-CAG-LSL-EGFP-bGH virus was used for conditional anterograde tracing in SERT^Cre/+^ mice. Injection was done at P4–5 and histology 3 weeks after. **b** Effective recombination is visible in PFC neurons that express EGFP (indicated by arrows). **c-e** Main brain targets of PFC-SERT+ neuron axons: **c** Thalamic nuclei including the paraventricular (PV), mediodorsal (MD), rhomboid (Rh), ventromedial (VM), and ventrolateral (VL) nuclei; (**d**) the substantia nigra pars compacta (SNpc) and ventral tegmental area (VTA); (**e**) the dorsal raphe nucleus (DRN) and periaqueductal gray (PAG). Laterodorsal nucleus (LD), aqueduct (Aq), substantia nigra pars reticulata (SNpr). **f** AAV1-CAG.FLEX-TdTomato-WPRE-bGH was bilaterally injected in the PFC of P4-P5 SERT^Cre/+^ mice used for the array tomographic anterograde tracing analysis in the DRN. **g** Array tomography image of seven serial-ultrathin 70-nm sections immunolabeled against synapsin (green), VGLUT1 (blue), and TdTomato (red), after anterograde viral tracing from the PFC. Asterisks indicate the same PFC TdTomato-positive axon terminal across the multiple serial sections often colabeled for VGLUT1 and synapsin. The arrow indicates a cortical axon bouton negative for TdTomato. The pie chart shows the percentages of cortical axon boutons present in the DRN that exclusively originated from PFC-SERT neurons (67 ± 8 %, red) in comparison with those arising from other cortical neurons (33 ± 8 %, blue)

To identify the projection targets of PFC-SERT+ neurons, we used conditional anterograde viral tracing coupled to immunohistochemical enhancement of axon labeling (Fig. 2a, b). To this end, we injected *AAV2/1-CAG-LSL-EGFP-bGH* in the PFC of P4-P5 SERT^Cre/+^ mice. This selectively labeled the SERT+ neurons in layers 5–6 of the PFC, including the infralimbic, prelimbic, and orbital regions (Fig. 2a, b). The identification of fibers of passage and axon terminals was based on their morphology (i.e., straight vs. varicose morphology, respectively). The distribution of axonal terminals across different brain regions allowed identifying their main neuroanatomical targets (Supplementary Figure 4). Most of the PFC-SERT+ neuron projections were directed to subcortical targets, including the medial thalamic cell groups (Fig. 2c), the lateral hypothalamus, and the brainstem. In the midbrain and hindbrain, a conspicuous innervation of the main monoaminergic nuclei was found in the ventral tegmental area and substantia nigra (Fig. 2d), the DRN (Fig. 2e), and to a lesser extent in the locus coeruleus. Comparison with previous detailed studies of the PFC projections arising from the prelimbic, infralimbic, and orbital regions [48, 49] indicated that PFC-SERT+ neurons belong to a specific subset within the broad PFC projections. Indeed, compared with these previous studies, PFC-SERT + neurons do not show callosal projections and only send minor projections to the amygdala, both areas that are classically innervated by PFC layers 5–6 neurons [48–50]. These findings indicate that Slc6a4/SERT expression identifies a subset of neurons within each of two broad subclasses of corticofugal pathways: the corticothalamic projections from layer 6 neurons and a sub-population of subcerebral projections from layer 5 neurons [51, 52].  Overall, the selective regional distribution of the SERT+ neurons within deep layers of the PFC suggests a main role in limbic neural circuits involved in top–downregulation of emotional control.

### PFC-SERT+ neurons are largely engaged in PFC-to-DRN circuits

Among the descending PFC pathways involving SERT+ neurons, the PFC-to-DRN circuit appeared to be an interesting candidate for further exploration, as it has a well-established role in stress and mood control [18–20]. Moreover, because the DRN has been shown to receive cortical inputs almost exclusively from the PFC [49, 50, 53], this allows quantitative input-specific analyses at the synaptic level. Interestingly, the topographic distribution of the PFC-to-DRN projection neurons shown in previous anatomical tracing studies is very similar to the distribution of the PFC-SERT+ neurons [49, 50, 54]. To determine the extent to which the PFC-SERT+ neurons contribute to the PFC-to-DRN circuit, we used anterograde viral tracing combined with a high-resolution immunofluorescence technique, array tomography, which allows quantitative analysis of the synaptic neuropil in volumes of brain tissue [31, 32, 34]. Using this approach, we estimated the proportion of PFC-SERT+ synaptic boutons within the total population of cortical synaptic afferents to the DRN. AAV-CAG.FLEX-Td-Tomato-WPRE-bGH was injected bilaterally into the PFC of SERT^Cre/+^ mice at P4–P5 to obtain a conditional TdTomato expression in a majority of the PFC-SERT+ neurons (Fig. 2f). A protein marker of cortical glutamatergic axon boutons, the vesicular glutamate transporter type 1 (VGLUT1) [55], was used to identify cortical terminals. Synapsin co-labeling was used as a general marker for synaptic boutons [34]. We analyzed the proportion of cortical synaptic boutons (VGLUT1+/synapsin+) that were also anterogradely labeled with TdTomato in the DRN using array tomography (Fig. 2g). All the TdTomato-labeled axon boutons contained VGLUT1 and 67% ± 8.0 % of the VGLUT1+/synapsin+ terminals were colabeled with TdTomato, indicating that these synaptic afferents arise from the PFC-SERT+ neurons (Fig. 2g). Overall, this experiment indicated that the PFC-SERT+ neuron population provides the majority of cortical inputs to the DRN and contain VGLUT1.

### Maturation of cortical axon projections to DRN parallels PFC Slc6a4/SERT expression

To explore the role of Slc6a4/SERT expression in the PFC-SERT+ neuron circuit assembly, we asked whether the timing of PFC Slc6a4/SERT expression corresponds to the development of PFC-DRN projections. To label all descending cortical projections, we took advantage of a mouse line that conditionally expresses TdTomato in all cortical glutamate projection neurons (i.e., EMX1b^Cre^:TdTomato). We then determined the timing of arrival of cortical axons to the DRN by directly measuring the ontogenetic increase of fluorescence within this structure. Quantitative evaluation of cortical axon arrival over the entire period of postnatal development (Supplementary Figure 5a) showed that while sparse cortical axons were detectable in the DRN at P4, their density increased about twofold between P4 and P14, a time when they reached about 80% of P21 values (Supplementary Figure 5a). Furthermore, western blot measurements in the DRN of VGLUT1 showed a significant increase in VGLUT1 protein levels over the first two postnatal weeks, with the maximal increase between P7 and P14 (Supplementary Figure 5b).

Collectively, these findings indicated an overlap in the dynamics of PFC Slc6a4/SERT expression and development of the PFC-to-DRN pathway, supporting the hypothesis that Slc6a4/SERT could play a role in the axon/synaptic maturation of this circuit.

### Lack of SERT during a developmental critical period results in synaptic hyperinnervation of the PFC-to-DRN circuit

**Fig. 3:**
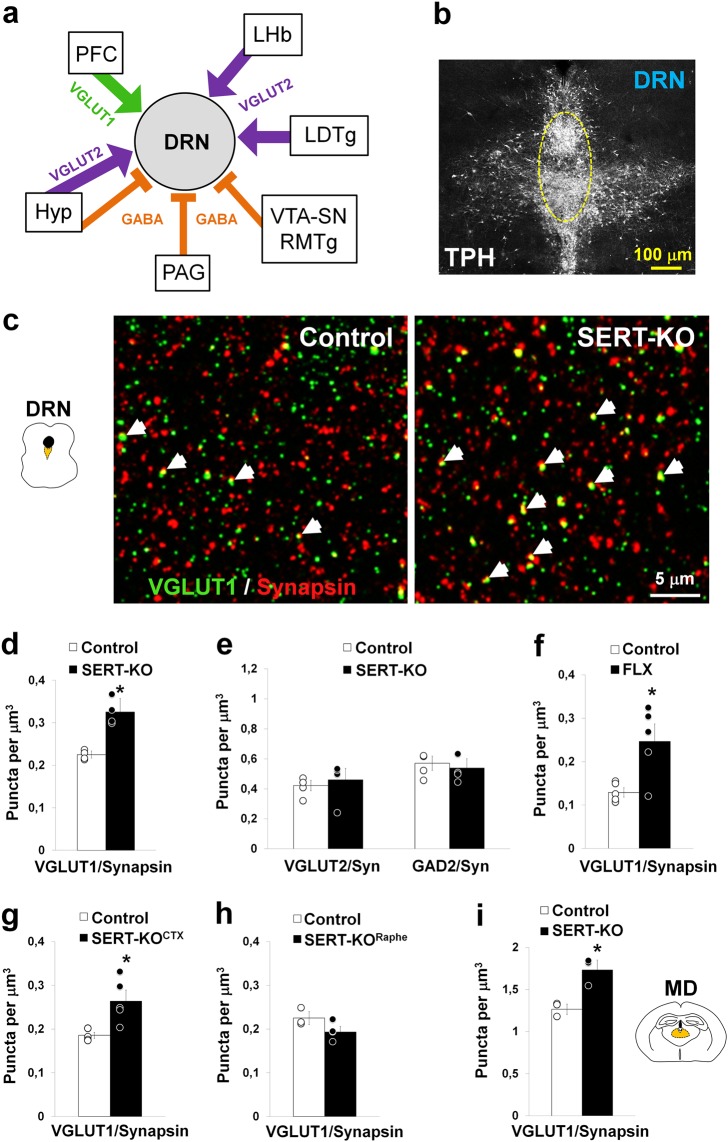
Cortical deletion of SERT results in synaptic hyperinnervation of the DRN. **a** Diagram summarizing the excitatory glutamate and inhibitory GABAergic synaptic inputs received by the dorsal raphe nucleus (DRN) neurons. Synaptic inputs can be selectively identified in array tomography by the presence of specific synaptic markers including the vesicular glutamate transporter type 1 and 2 (VGLUT1 and VGLUT2, respectively) and the enzyme responsible for GABA synthesis, the glutamate decarboxylase 2 (GAD2). The prefrontal cortex (PFC), lateral habenula (LHb), laterodorsal tegmental nucleus (LDTg), ventral tegmental area (VTA), substantia nigra (SN), rostromedial tegmental nucleus (RMTg), periaqueductal gray (PAG), and hypothalamus (Hyp), have been noted as the main synaptic inputs to the DRN [50, 53, 54]. **b** Immunolabeling against the 5-HT biosynthetic enzyme tryptophan hydroxylase (TPH) illustrating the distribution of 5-HT neurons in the midbrain DRN. The bracketed area shows the sampling region in the midline DRN used for array tomography quantitative analyses (at P28). **c** Array tomography projection image of three serial-ultrathin 70-nm-thick sections of the DRN immunolabeled against VGLUT1 (green) and synapsin (red) to specifically identify cortical synaptic boutons. The arrows indicate double-labeled boutons in control and SERT-KO mice. **d-e** Quantitative analysis of cortical glutamate synaptic boutons (VGLUT1+) (**d**), and subcortical glutamate (VGLUT2+) and GABAergic (GAD2+) synaptic boutons (**e**) in the DRN of control and SERT-KO mice (4 mice/genotype; for VGLUT1/Synapsin pairs: F_1,6_ = 36.45, *p < 0.001; for VGLUT2/Synapsin pairs: F_1,6_ = 0.24, *p* = 0.64; and for GAD2/Synapsin pairs: F_1,6_ = 0.34, *p* = 0.58). **f** Analysis of cortical synaptic boutons in the DRN after fluoxetine-treatment (FLX) during the postnatal critical period (P2-14) (5 mice/group; F_1,8_ = 9.94, *p < 0.02). **g-h** Density of cortical synaptic boutons in the DRN after conditional SERT invalidation using Emx1b^Cre/+^:Sert^fl/fl^ mice (SERT-KO^CTX^) (**g**) and Pet1^Cre^:Sert^fl/fl^ mice (SERT-KO^Raphe^) (**h**). (**g):** 5 mice/genotype (Welch’s statistic = 11.20, *p < 0.03); (**h**): 3–4 mice/genotype (F_1,5_ = 3.76, *p* = 0.11). **i** Cortical synaptic boutons in the mediodorsal thalamic nucleus (MD) of control and SERT-KO mice (3 mice/genotype; F_1,4_ = 18.16, *p < 0.02). One-way ANOVA (**d, e, f, h, i**), and Welch’s *t* test (**g**). Error bars represent SEM

To investigate whether Slc6a4/SERT has a role in axon/synapse development of the PFC-to-DRN circuit, we used array tomography to determine if lack of Slc6a4/SERT expression (SERT-KO) modifies the density of cortical synaptic afferents on DRN neurons (Fig. 3a, b). Because anterograde tracing experiments are inherently variable, making quantitative comparisons between genotypes arduous, we relied instead on VGLUT1 immunolabeling. VGLUT1 labels only cortical afferents in the brainstem [55, 56], the majority of which arise from deep cortical layers of the PFC [50, 53, 54], where SERT is transiently expressed. We found that SERT-KO mice have a 40% increase in the number of VGLUT1+ axon boutons in the DRN (Fig. 3c, d) compared to SERT^+/-^ mice at 4 weeks of age. To determine whether this increase in synaptic innervation is specific to the VGLUT1+ glutamatergic inputs arising from the cortex (Fig. 3a), we examined the other sources of excitatory glutamatergic inputs to the DRN. These arise from subcortical sources and are labeled with the vesicular glutamate transporter type 2 (VGLUT2). Additionally, we analyzed the inhibitory GABAergic synaptic inputs to the DRN, using glutamate decarboxylase 2, (GAD2) as a marker [34] (Fig. 3e). This analysis showed that VGLUT2+ and GAD2+ synaptic inputs were unchanged in the SERT-KO, indicating a high degree of specificity of changes for the VGLUT1+ synapses.

Interestingly, a similar increase in the number of VGLUT1+ synapses was observed in the corticothalamic (PFC-to-MD) circuit (Fig. 3i), which largely derives from the PFC-SERT+ neurons (Supplementary Figure 4), further suggesting that this Slc6a4/SERT mechanism could be valid for other corticofugal circuits in which PFC-SERT+ neurons are engaged. Conversely, in the basolateral nucleus of the amygdala (BLA), where PFC-SERT+ terminals are rare (Supplementary Figure 4), we observed no change in the density of VGLUT1+ synapses (Supplementary Fig. 6l), suggesting that SERT blockade has a selective impact on the maturation of the PFC-SERT+ neurons, although we cannot exclude other developmental effects on other PFC neurons.

Next, we examined whether transient pharmacological blockade of Slc6a4/SERT function with SSRIs during the critical postnatal period could reproduce these effects. Wild-type mice were treated with fluoxetine (per os, 10 mg/kg/day) or sucrose from P2 to P14 [4, 6], and we then examined the density of PFC-to-DRN synaptic innervations in comparison to vehicle-treated mice. These experiments showed that postnatal fluoxetine induces a 47% increase in the number of VGLUT1+ cortical synaptic innervations in the DRN (Fig. 3f) without affecting the other subcortical glutamatergic (VGLUT2+) or GABAergic (GAD2+) inputs (Supplementary Figure 6d), as in the SERT-KO mice.

Since the PFC-to-DRN projection targets both the 5-HT and GABA neuronal populations [32, 53], we examined whether changes of synaptic innervation caused by Slc6a4/SERT loss-of-function could differentially impact 5-HT neurons of the DRN. For this, we analyzed VGLUT1+ axon boutons in conjunction with immunolabeling for the enzyme tryptophan hydroxylase (TPH) to identify 5-HT neurons [34]. Analyses in SERT-KO- and fluoxetine-treated mice showed an increased number of VGLUT1+ synapses on 5-HT neurons (Supplementary Fig. 6a, c), while other glutamate (VGLUT2+) and GABAergic (GAD2+) synaptic afferents impinging onto 5-HT neurons were not affected (Supplementary Fig. 6b, e). Overall, these results show that transient blockade of Slc6a4/SERT function during the postnatal period is sufficient to increase synapse formation in the PFC-to-DRN circuits. Because 50% of VGLUT1+ synapses are associated to 5-HT neurons this suggests that these modifications occur on both the 5-HT and non-5-HT cell populations of the DRN.

### Cell-autonomous role of Slc6a4/SERT in the synaptic wiring of the PFC-to-DRN circuit

 Slc6a4/SERT is expressed broadly in the brain during development, including the raphe nuclei and several other brain regions [21, 42, 44]. To determine the role of the cortical or raphe Slc6a4/SERT we used a conditional genetic approach. The recently developed SERT floxed mice (SERT^fl/fl^) [28, 29] were crossed with the EMX1b^Cre^ mice that drives Slc6a4/SERT invalidation in all cortical projection neurons (SERT^fl/fl^:EMX1b^Cre^ = SERT-KO^CTX^). Array tomography analyses in the DRN of the SERT-KO^CTX^ mice at 4 weeks of age showed a 30% increase in the number of VGLUT1+ synaptic boutons (Fig. 3g). Conversely, conditional invalidation of SERT in the raphe 5-HT neurons (SERT^fl/fl^:Pet1^Cre^ = SERT-KO^Raphe^) did not change the density of VGLUT1+ synapses in the DRN (Fig. 3h). Both SERT-KO^CTX^ and SERT-KO^Raphe^ mice showed no compensatory alterations of VGLUT2+ subcortical glutamatergic and GAD2+ GABAergic synaptic innervations (Supplementary Fig. 6g, j). These findings indicate that a cortex-specific Slc6a4/SERT-dependent mechanism is involved in the synaptic wiring of this circuit.

As in the SERT-KO, we determined whether conditional deletion of Slc6a4/SERT from the cortex or raphe modifies the density of glutamate and GABAergic synapses onto 5-HT neurons. An increase in the density of cortical VGLUT1+ synaptic afferents onto 5-HT neurons was noted in the SERT-KO^CTX^ (Supplementary Fig. 6f), but not in the SERT^Raphe^ mice (Supplementary Fig. 6i). These changes were specific of cortical synapses, since VGLUT2+ or GABAergic (GAD2+) synaptic boutons were not affected by any of the strategies of Slc6a4/SERT invalidation (Supplementary Fig. 6h, k).

Overall, these findings indicate a cell-autonomous role of cortical Slc6a4/SERT for increasing synapse formation in the PFC-to-DRN circuit.

### Lack of SERT increases the number of functional PFC-to-DRN synapses

**Fig. 4:**
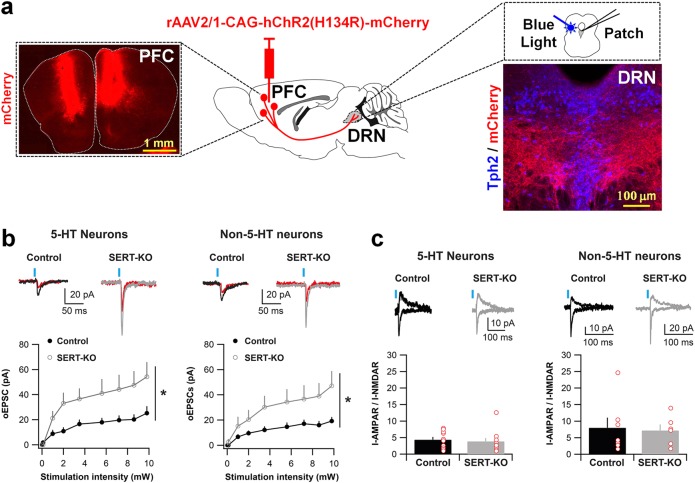
Lack of SERT increases the number of functional PFC-to-DRN synapses. **a** rAAV-CAG-hChR2(H134R)-mCherry was bilaterally injected into the PFC of P4–P5 control or SERT-KO mice. Photograph showing mCherry expression after the PFC AAV injection (upper left). Optogenetic stimulation and electrophysiological patch clamp recordings were made starting at P28 in coronal sections containing the DRN, as shown by the photograph of the immunolabeling of PFC mCherry+ axons innervating to DRN 5-HT neurons, identified by the presence of the enzyme TPH2 (upper right). **b** Amplitude of optogenetically evoked EPSCs (oEPSCs) at synapses from PFC terminals onto DRN putative 5-HT neurons (left) and non-5-HT neurons (right) at various light stimulation intensities. In control (SERT^Cre/+^) (5-HT: *n* = 10 cells/5 animals; non-5-HT: *n* = 7 cells/4 animals); in SERT-KO (SERT^Cre/Cre^) (5-HT: *n* = 10 cells/3 animals; non-5-HT: *n* = 6 cells/3 animals). Top: example traces at 9.8 mW (black/gray) and at 2 mW (red) stimulation); Bottom: input/output curves. Two-way ANOVA on 9.8 mW intensity: genotype x cell-type interaction (F_1,29_ = 0.003, *p* = 0.95); Genotype main effect (F_1,29_ = 9.32, *p < 0.01); Cell-type main effect (F_1,29_ = 0.51, *p* = 0.48). **c** AMPAR/NMDAR ratios at synapses from PFC-to-DRN 5-HT neurons (left) and non-5-HT neurons (right) in control (5-HT: *n* = 10 cells/4 animals; non-5-HT: *n* = 7 cells/3 animals), and SERT-KO (5-HT: *n* = 11 cells/3 animals; non-5-HT: *n* = 6 cells; 3 animals). The AMPAR responses were calculated at the peak of −50 mV, whereas NMDAR responses were determined at + 40 mV, 50 ms after stimulation. Top: example traces; bottom: bar graphs. Two-ways ANOVA: Genotype x Cell-type interaction (F_1,30 = _0.007, *p* = 0.94); Genotype main effect (F_1,30_ = 0.16, *p* = 0.69); Cell-type main effect (F_1,30_ = 4.51, *p* < 0.05). Blue bars indicate blue light stimulation. Error bars represent SEM

To determine the impact of increased PFC-to-DRN synapse formation on the excitatory synaptic drive in the DRN, we studied glutamate excitatory transmission in the SERT-KO mice using ex vivo patch clamp electrophysiology and optogenetically driven PFC axon terminal stimulation. For this, rAAV-CAG-hChR2(H134R)-mCherry was bilaterally injected in the PFC of SERT-KO and SERT^+/-^ mice at P4–P5 (Fig. 4a). After 3 weeks, we performed in vitro electrophysiological recordings in DRN neurons identifying putative 5-HT and GABA types based on their distinctive cellular properties [34, 35]. The identity of the recorded cells was corroborated by post hoc immunochemical analysis combining Alexa 488 electroporation of the recorded cells with immunohistochemistry against the 5-HT biosynthetic enzyme tryptophan hydroxylase (Supplementary Fig. 7). Optical stimulation of the PFC-to-DRN synapses in SERT-KO mice expressing ChR2 led on average to larger AMPA receptor (AMPAR)-mediated excitatory postsynaptic currents (EPSCs) when compared with control animals, and this effect was present on both putative 5-HT and GABA neurons, as identified by electrophysiological criteria (Fig. 4b). We observed no modification in the AMPA-to-NMDA ratios in any of the two types of DRN neurons recorded (Fig. 4c). This suggests that postsynaptic properties of PFC-to-DRN synapses remain unaffected in the SERT-KO mouse. Instead, these data are in accordance with the presence of an increased number of functional glutamatergic synapses from the PFC onto both 5-HT and non-5-HT neurons in the DRN of SERT-KO mice, consistent with our array tomography study. Taken together, these findings provided strong evidence supporting a role of Slc6a4/SERT in controlling the glutamatergic synaptic drive of the PFC-to-raphe circuit.

### Bidirectional modulation of the emotional deficits induced by early postnatal fluoxetine upon stimulation or inhibition of the PFC-SERT+ neurons

**Fig. 5:**
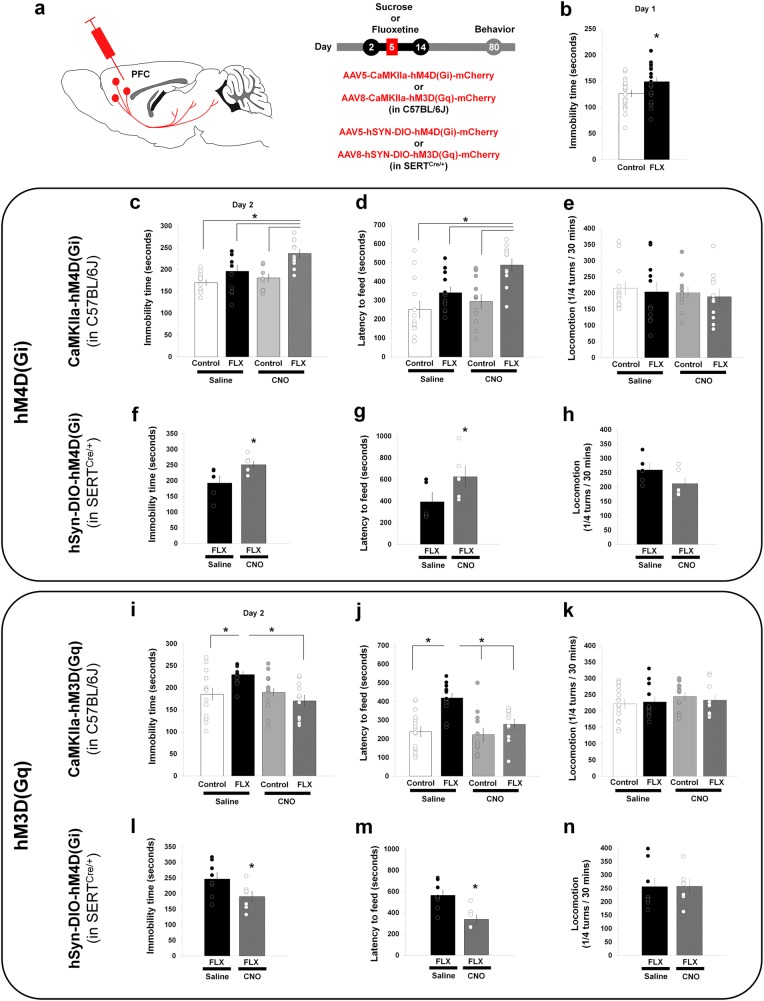
Bidirectional modulation of developmental SSRI-induced emotional alterations by PFC-SERT+ neurons. **a** For chemogenetic manipulation of PFC glutamate projection-neurons, AAV5-CaMKIIa-hM4D(Gi)-mCherry (**c-e**) or AAV8-CaMKIIa-hM3D(Gq)-mCherry (**i**-**k**) was bilaterally injected into the PFC of P5 wild-type C57BL/6 mice. For conditional expression in PFC-SERT neurons the AAV5-hSYN-DIO-hM4D(Gi)-mCherry (**f-h**) and AAV8-hSYN-DIO-hM3D(Gq)-mCherry (**l-n**) were used in SERT^Cre/+^ mice. Mice were treated with fluoxetine (FLX) (10 mg/kg/day in 5% sucrose) or with 5% sucrose (SUC), from P2 to P14. Behavioral analyses were started at P80, starting with the novelty-suppressed feeding test (NSF), the forced swim test (FST), and locomotor activity spaced by 7 days intervals. **b** Immobility time in the FST during the first day “drug-free” session. Control group: 25 mice (15 males, 10 females); FLX group: 23 mice (10 males, 13 females). Two-ways ANOVA: gender x treatment interaction (F_1,44_ = 1.855, *p* = 0.18); gender main effect (F_1,44_ = 6.407, *p* < 0.02); treatment main effect (F_1,44_ = 5.228, *p < 0.03). **c**: Immobility time in the FST (day 2); **d**: latency to feed in the NSF; **e:** locomotor activity, after a single i.p. injection of saline (NaCl 0.9%) or CNO (1 mg/kg) 30 min. before the testing. Two-ways ANOVA of the FST (**c**): treatment combination x gender interaction (F_3,40_ = 1.345, *p* = 0.27); treatment combination main effect (F_3,40_ = 9.510, *p* < 0.0001); gender main effect (F_1,40_ = 3.550, *p* = 0.07). FLX-CNO vs. Control-Saline (*p < 0.0001), vs. Control-CNO (*p < 0.001) and vs. FLX-Saline (*p < 0.02) by Tukey’s test. Two-ways ANOVA of NSF (d): treatment combination x gender interaction (F_3,40_ = 0.657, *p* = 0.58); treatment combination main effect (F_3,40_ = 6.994, *p* < 0.001); gender main effect (F_1,40_ = 0.011, *p* = 0.916). FLX-CNO vs. Control-Saline (*p < 0.001), vs. Control-CNO (*p < 0.004) and vs. FLX-Saline (*p < 0.04) by Tukey’s test. Two-ways ANOVA of locomotor (**e**): treatment combination x gender interaction (F_3,40_ = 0.245, *p* = 0.865); treatment combination main effect (F_3,40_ = 0.394, *p* = 0.758); gender main effect (F_1,40_ = 3.318, *p* = 0.08). In (**c-e**): Control/Saline group: 13 mice (8 males, 5 females); Control/CNO group: 12 mice (7 males, 5 females); FLX/Saline group: 12 mice (6 males, 6 females); FLX/CNO group: 11 mice (4 males, 7 females). **f-h** Conditional inhibition of PFC-SERT neurons in SERT^Cre/+^ mice treated or not with fluoxetine (FLX) from P2 to 14) (**a**). Immobility in the FST (day 2) (**f**), latency to feed in the NSF (**g**) and locomotor activity (**h**) after an i.p. injection of either CNO (1 mg/kg) or NaCl 0.9% saline (SAL), 30 min. before testing. In (**f**): ANOVA: treatment main effect (F_1,9_ = 5.982, *p* < 0.04), in (**g**): ANOVA: treatment main effect (F_1,9_ = 6.287, *p* < 0.04), in (**h**): ANOVA: treatment main effect (F_1,9_ = 2.712, *p* = 0.134). In (**f-h**): FLX/Saline group: 5 males; FLX/CNO group: 6 males. Immobility time in the FST (day 2) (**i**), latency to feed in the NSF (**j**) and locomotor activity (**k**), after a single i.p. injection of either CNO (1 mg/kg) or NaCl 0.9% saline, 30 min. before testing. Two-ways ANOVA in (i): treatment combination x gender interaction (F_3,43_ = 0.612, *p* = 0.61); treatment combination main effect (F_3,43_ = 4.177, *p* < 0.02); gender main effect (F_1,43_ = 0.315, *p* = 0.58). FLX-Saline vs. control-saline (*p < 0.05), vs. FLX-CNO (*p < 0.01) by Tukey’s test. Two-ways ANOVA in (j): treatment combination x gender interaction (F_3,43_ = 0.490, *p* = 0.69); treatment combination main effect (F_3,43_ = 9.532, *p* < 0.0001); gender main effect (F_1,43_ = 3.218, *p* = 0.08). FLX-Saline vs. Control-Saline (*p < 0.0002), vs. Control-CNO (*p < 0.0001) and vs. FLX-CNO (*p < 0.01) by Tukey’s test. Two-ways ANOVA in (k): treatment combination x gender interaction (F_3,43_ = 0.815, *p* = 0.492); treatment combination main effect (F_3,43_ = 0.521, *p* = 0.670); gender main effect (F_1,43_ = 0.137, *p* = 0.713). In (**i-k**): Control/Saline group: 16 mice (8 males, 8 females); Control/CNO group: 12 mice (8 males, 4 females); FLX/Saline group: 12 mice (6 males, 6 females); FLX/CNO group: 11 mice (4 males, 7 females). **l-n** Conditional activation of PFC-SERT neurons in SERT^Cre/+^ mice treated or not with fluoxetine (FLX) from P2 to P14). **a** Immobility in the FST (day 2) (**l**), latency to feed in the NSF (**m**) and locomotor activity (**n**) after an i.p. injection of either CNO (1 mg/kg) or NaCl 0.9% saline (SAL), 30 min. before the testing. In (**l**): ANOVA: treatment main effect (F_1,13_ = 5.100, *p* < 0.05), in (**m**): ANOVA: treatment main effect (F_1,13_ = 13.792, *p* < 0.01), in (**n**): ANOVA: treatment main effect (F_1,13_ = 0.0006, *p* = 0.981). In (**l-n**): FLX/Saline group: 8 males; FLX/CNO group: 7 males

SERT-KO mice show increased passive coping strategies when exposed to an acute stressor: they float more in the forced swim test (FST) and have an increased latency to feed in the novelty-suppressed feeding test (NSF). These behavioral alterations are reproduced by early-life exposure to SSRIs, suggesting that they have a developmental origin [4, 6, 8]. Since the PFC exerts a top–down control in stress-coping responses [18, 19, 57], we reasoned that increased innervation of the PFC descending circuits could underlie the adult emotional deficits induced by early-life SSRIs. To test this, we used a pharmacogenetic approach [30] to either decrease or increase the activity of the PFC-SERT+ neurons in adult mice that had been exposed to SSRIs during early postnatal life. Cohorts of wild-type mice were treated with either fluoxetine (per os, 10 mg/kg/day) or 5% sucrose from P2 to P14 (Fig. 5a). All mice (fluoxetine-treated or sucrose-treated) were bilaterally injected in the PFC as pups with either AAV5-CaMKIIa-hM4D(Gi)-mCherry or AAV8-CaMKIIa-hM3D(Gq)-mCherry. Post hoc histological controls showed that the virus was efficiently transduced only in PFC pyramidal neurons and mainly in layer 5–6 of the prelimbic, infralimbic and orbital regions (Supplementary Fig. 8a, d). The efficiency of CNO in inhibiting or activating the activity of the PFC glutamate projection neurons was controlled. In mice injected with either AAV5-CaMKIIa-hM4D(Gi)-mCherry or AAV8-CaMKIIa-hM3D(Gq)-mCherry, the CNO (1 mg/kg) treatment either reduced by 80% or increased above two-fold c-Fos expression in the transduced pyramidal neurons (hM4D(Gi)-mCherry+ and hM3D(Gq)-mCherry+, respectively) thus validating the approach (Supplementary Fig. 8b, c).

Adult mice that had been exposed to fluoxetine during early life showed increased floating behavior in the FST (Fig. 5b, c, i) and increased latency to feed in the NSF compared with control mice (Fig. 5d, j), consistent with previous studies [4, 6]. Similar effects were observed in both genders, although differences in the magnitude of the effects between genders were found in the FST on day one, differences did not reach significance for the FST at day two nor for the NSF or locomotor behavior. This showed that our early viral injections of DREADDs into the PFC did not interfere with the known effects of early postnatal fluoxetine on adult emotional behaviors. We then examined, in mice injected with AAV5-CaMKIIa-hM4D(Gi)-mCherry, whether acute inhibition of the PFC pyramidal neurons impacts behavioral outputs. In the control sucrose-treated group, CNO administration 30 min before the testing had no effect in the FST (Fig. 5c**)** and the NSF (Fig. 5d), indicating that inhibition of the PFC glutamate projection-neurons does not alter *per se* these behavioral responses. However, in the fluoxetine-treated group, CNO administration increased both the immobility time in the FST (Fig. 5c) and the latency to feed in the NSF (Fig. 5d), without changing spontaneous locomotor responses (Fig. 5e). We then tested the effects of acute activation in mice injected with AAV8-CaMKIIa-hM3D(Gq)-mCherry. In these mice, CNO administered before the behavioral test reduced the immobility time in the FST (Fig. 5i) and decreased the latency to feed in the NSF(Fig. 5j), without locomotor changes (Fig. 5k). This effect was only present in the fluoxetine-treated group. These results indicated that silencing of adult PFC glutamate neurons enhances the SSRI-induced emotional alterations, while activation rescues these behavioral deficits.

As these bidirectional modulatory effects might be due to indiscriminate  recruitment of different subtypes of PFC pyramidal neurons, we conditionally restricted the hM4D(Gi) and hM3D(Gq) expression to the SERT+ projection-neurons. Hence, we repeated these experiments in SERT^Cre/+^ mice exposed to fluoxetine during early life using a conditional virus expressing hM4D(Gi) or hM3D(Gq) (Fig. 5a). Consistent with our previous experiments, acute inhibition of SERT+ neuron’s activity with CNO increased both the immobility time in the FST (Fig. 5f) and the latency to feed in the NSF (Fig. 5g) without changing locomotor activity (Fig. 5h). Conversely, CNO activation of SERT+ neurons decreased the immobility time in the FST (Fig. 5l) and the latency to feed in the NSF (Fig. 5m) without locomotor changes (Fig. 5n). These experiments showed that selective inhibition or activation of PFC-SERT+neurons was sufficient to reproduce the behavioral effects observed after a more general manipulation of PFC neuronal activity, namely increasing activity of the PFC-SERT+ neurons rescues the anxiety and depresssion-like responses caused by postnatal fluoxetine administration.

Overall, these results suggest that the increased synaptic drive from the PFC induced by early-life exposure to SSRIs acts to mitigate passive stress-coping responses, and thus counterbalancing the negative effects of SSRI exposure on emotional behavior.

## Discussion

Our study identified a corticofugal pathway—the PFC-to-DRN circuit—that is modulated by 5-HT-related antidepressants during a critical developmental period corresponding to the phase of active synaptogenesis. Transient expression of Slc6a4/SERT in a subset of sub-cortically projecting pyramidal neurons of the PFC controls glutamatergic synapse formation in the thalamus and raphe likely by influencing expression of genes involved in axon growth/synapse formation. These findings provide novel insights into how genetic and environmental perturbations of 5-HT transmission during early life can lead to significant changes in the assembly of specific PFC circuits with long-lasting consequences on emotional behavior.

Altered development of PFC circuits is thought to underlie several psychiatric disorders, including anxiety/depression-related disorders [58]. Previous histological studies reported changes of cell density and layer thickness in the cortex [59] and dendritic changes in the infralimbic PFC [11] of SERT-KO mice. However, it has been difficult to pinpoint-specific PFC circuits that could be selectively altered by early developmental events due to both the complexity of the PFC circuits, and the lack of precise knowledge about their development [60].

Our study demonstrates that a major output connection of the PFC directed to the brainstem shows a critical period during which its synaptic wiring is tightly controlled by changes of 5-HT transmission. Indeed, the full or cortex-specific invalidation of Slc6a4/SERT results in synaptic hyperinnervation of the PFC-to-DRN circuit. Furthermore, this abnormal synaptic wiring is reproduced by the pharmacological administration of the selective Slc6a4/SERT blocker fluoxetine from P2 to P14. The critical period for this effect coincides with the developmental period where early stressors and exposure to SSRIs have been found to have long-lasting effects on anxiety- and depressive-like behaviors, as well as on cognitive PFC function [6, 7, 61]. The PFC output circuits to subcortical brain centers are important modulators of mood in particular via top–down control of monoaminergic brainstem nuclei such as the dopamine neurons in the VTA [62] and the 5-HT raphe neurons [53, 63]. Preclinical and clinical studies indicated that the PFC-to-DRN circuit plays a crucial role in stress-controllability [18, 19]. Stimulation of this pathway has an antidepressant effect in rats [19]. Moreover, in humans, this circuit is thought to underlie some of the beneficial effects of deep brain stimulation in depression [16, 64]. Thus, developmental synaptic miswiring of this circuit is likely to have an important impact on the pathophysiology of mood-related disorders. However, since synaptic plasticity is a dynamic process continuing in adult life, it remains to be determined whether the increased number of functional synapses that we observed in juvenile animals persists throughout life.

We found that altering Slc6a4/SERT function during development selectively increases the number of PFC glutamatergic synapses in the DRN, without changing other subcortical glutamatergic or GABAergic synaptic inputs to raphe neurons. Moreover, the enhanced synapse number caused by SERT invalidation resulted in functionally increased glutamatergic transmission at PFC-to-DRN synapses; this could not be accounted for by postsynaptic changes in the AMPA/NMDA ratios. In the raphe, PFC synaptic inputs are directed to both 5-HT and non-5-HT GABAergic neurons [32, 53]. The latter play a crucial role in feed-forward inhibitory modulation of 5-HT neuron activity [20, 65] and on PFC glutamate terminals [34, 66]. Our observations indicate that when Slc6a4/SERT is invalidated genetically or pharmacologically, the supernumerary PFC synapses are not exclusively associated with 5-HT neurons but are also largely on non-5-HT neurons, possibly GABA neurons, thus being in a position to either enhance or inhibit 5-HT output. However, previous physiological studies argue for an overall increased inhibitory effect in the DRN upon transient SERT inactivation. First, in normal conditions the PFC has a main inhibitory effect on 5-HT neuron excitability [65, 67]. Second, in vivo electrophysiological studies in SERT-KO mice [8] and in mice exposed to SSRIs during early life showed a reduced excitatory tone of the 5-HT DRN neurons [68]. These observations are then consistent with the suggestion of an increased feed-forward inhibition from the PFC in conditions of early life SERT invalidation. However these studies were performed in anesthetized rodents, and such regulations are likely highly dynamic. Thus, an important  direction for future research will be to determine how these circuits function in behaving animals under different stress conditions.

Our experiments showed that activation of the PFC-SERT+ neurons in adult life rescued the anxiety/depressive-like phenotypes of mice exposed to SSRIs during early life, while inhibition of this neuronal population enhanced these phenotypes. Thus, at a system level, the hyperinnervation of PFC descending circuits would seem to counterbalance functional alterations within the PFC network. Based on these experiments, this suggests that developmental exposure to SSRIs results in a general hypoexcitability of PFC networks. One can only speculate at this point on the mechanisms involved which could involve intrinsic changes in the excitability of the PFC pyramidal neurons, or changes of the excitatory/inhibitory drive. Previous studies have shown that perinatal exposure to SSRIs impacts the development of several stress-related circuits [69, 70]. Thus, it will be interesting in future studies to tease apart the different factors that contribute to changes in the activity of PFC neurons after early life exposure to SSRIs.

The protracted development of the PFC makes it particularly vulnerable to the effects of environmental insults. Our observation that Slc6a4/SERT plays a role in the synaptic wiring of a large population of PFC neurons provides further evidence to understand these effects. It shows that enhancing 5-HT neurotransmission in the PFC modifies the transcriptional program of selected PFC neurons leading to enhance their axon growth and synaptogenic potential. At a cell-signaling level, the main function of Slc6a4/SERT in these PFC neurons could be to act as a buffer/sink to regulate extracellular levels of 5-HT. Indeed, based on transcriptome profiling results, and pharmacological manipulations, the PFC-SERT+ neurons are able to degrade 5-HT, but not to synthesize it, with MAOA playing a key role in the degradation of 5-HT. This clearance function is crucial to control the 5-HT levels locally in the cortex since only the cortex-specific but not the raphe-specific Slc6a4/SERT invalidation reproduced the effects of the full SERT-KO. Although some parallels can be drawn with the function of transient SERT expression in the thalamocortical and retinotectal systems, the mechanisms are likely to be different. In the barrel cortex and retinal system the main role of Slc6a4/SERT is to moderate the inhibitory effects of 5-HT1B receptors on neurotransmitter release [71, 72] and thereby to modulate activity-dependent mechanisms that shape precise topographic maps [73]. In the PFC, the signaling mechanisms that are associated to transient Slc6a4/SERT expression are likely different, since the 5-HT1B receptor is not expressed in the developing PFC neurons and also because the main effect observed is an increased synapse formation. Therefore, it will be interesting to investigate the role of other receptors that are expressed in the developing PFC, focusing on those, such as 5-HT2A and 5-HT7 that have been shown to promote synaptogenesis [74].

In a translational perspective it is noteworthy that similar mechanisms appear to exist in the human brain. On one side, evidence indicates that altered connectivity of PFC circuits contribute to mood disorders [12, 13]. On the other side, mounting evidence indicates that prenatal SSRI exposure contributes to anxious and internalizing behaviors in childhood [75, 76], and to depressive symptoms during adolescence [77]. Moreover, reduced Slc6a4/SERT functionality, as reported in human carriers of low-functioning Slc6a4/SERT alleles, has been linked to anxiety-related behavioral traits [78, 79] and vulnerability to childhood trauma and depression [80]. Finally, Slc6a4/SERT is transiently expressed in the human embryonic frontal cortex [44] **(**Supplementary Fig. 9; transcriptional data from Brainspan Atlas of the Developing Human Brain, http://www.brainspan.org), further supporting the notion of a common developmental target in humans.

Our findings underline the fact that important changes in prefrontal circuits, driven by SERT+ neurons can take place during critical developmental periods, in particular in shaping top–down prefrontal circuits to the brainstem that play a crucial role in adaptive behaviors to stress. Altered development of these circuits contributes to shape stress-coping strategies later in life.

## Electronic supplementary material

Supplementary Material

Supplementary table 1

supplementary table 4
