# Correction: SSRIs target prefrontal to raphe circuits during development modulating synaptic connectivity and emotional behavior

**DOI:** 10.1038/s41380-019-0349-9

**Published:** 2019-01-10

**Authors:** M. Soiza-Reilly, F. J. Meye, J. Olusakin, L. Telley, E. Petit, X. Chen, M. Mameli, D. Jabaudon, J.-Y. Sze, P. Gaspar

**Affiliations:** 1 Institut du Fer à Moulin, Paris, France; 2 Inserm, UMR-S 839, Paris, France; 3 Sorbonne Universités, Paris, France; 4 Department of Basic Neurosciences, University of Geneva, Geneva, Switzerland; 5 Department of Molecular Pharmacology, Albert Einstein College of Medicine, Bronx, New York, USA

**Keywords:** Neuroscience, Depression

**Correction to: Molecular Psychiatry**

10.1038/s41380-018-0260-9; published online: 02 Oct 2018

This article was originally published under standard licence, but has now been made available under a [CC BY 4.0] license. The PDF and HTML versions of the paper have been modified accordingly.

