# Supplementary material for: SSRIs target prefrontal to raphe circuits during development modulating synaptic connectivity and emotional behavior
Source: Mol Psychiatry. 2018 Oct 2;24(5):726–45. doi: 10.1038/s41380-018-0260-9 (PMC6445781; doi:10.1038/s41380-018-0260-9)
Supplement: Supplementary file 1 — Supplementary Material [file 41380_2018_260_MOESM1_ESM.pdf]

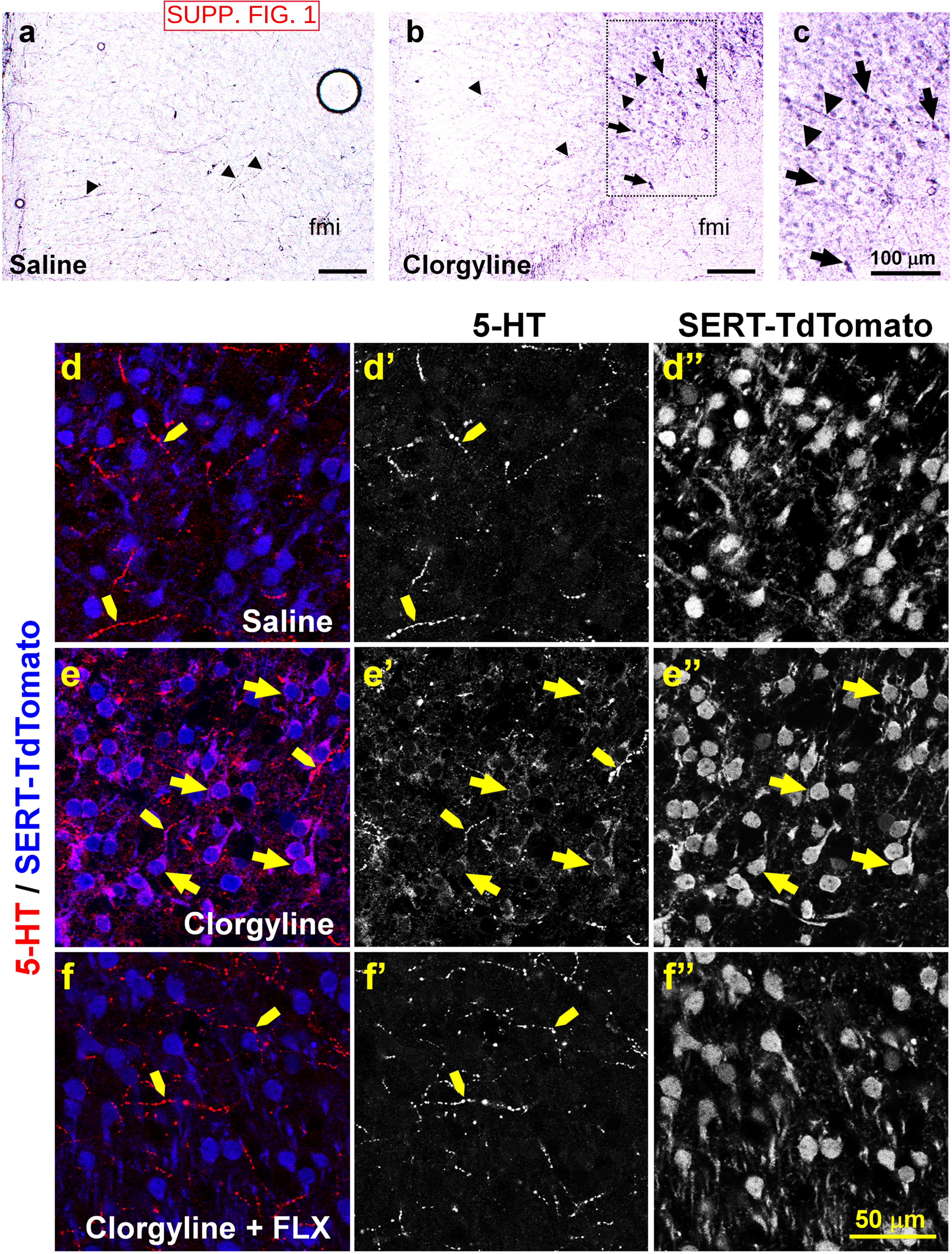
SUPP. FIG. 1
a
Saline
fmi
b
Clorgyline
fmi
c
100 µm
5-HT
SERT-TdTomato
5-HT / SERT-TdTomato
d
d'
d''
Saline
e
e'
e''
Clorgyline
f
f'
f''
Clorgyline + FLX
50 µm

SUPP. FIG. 2

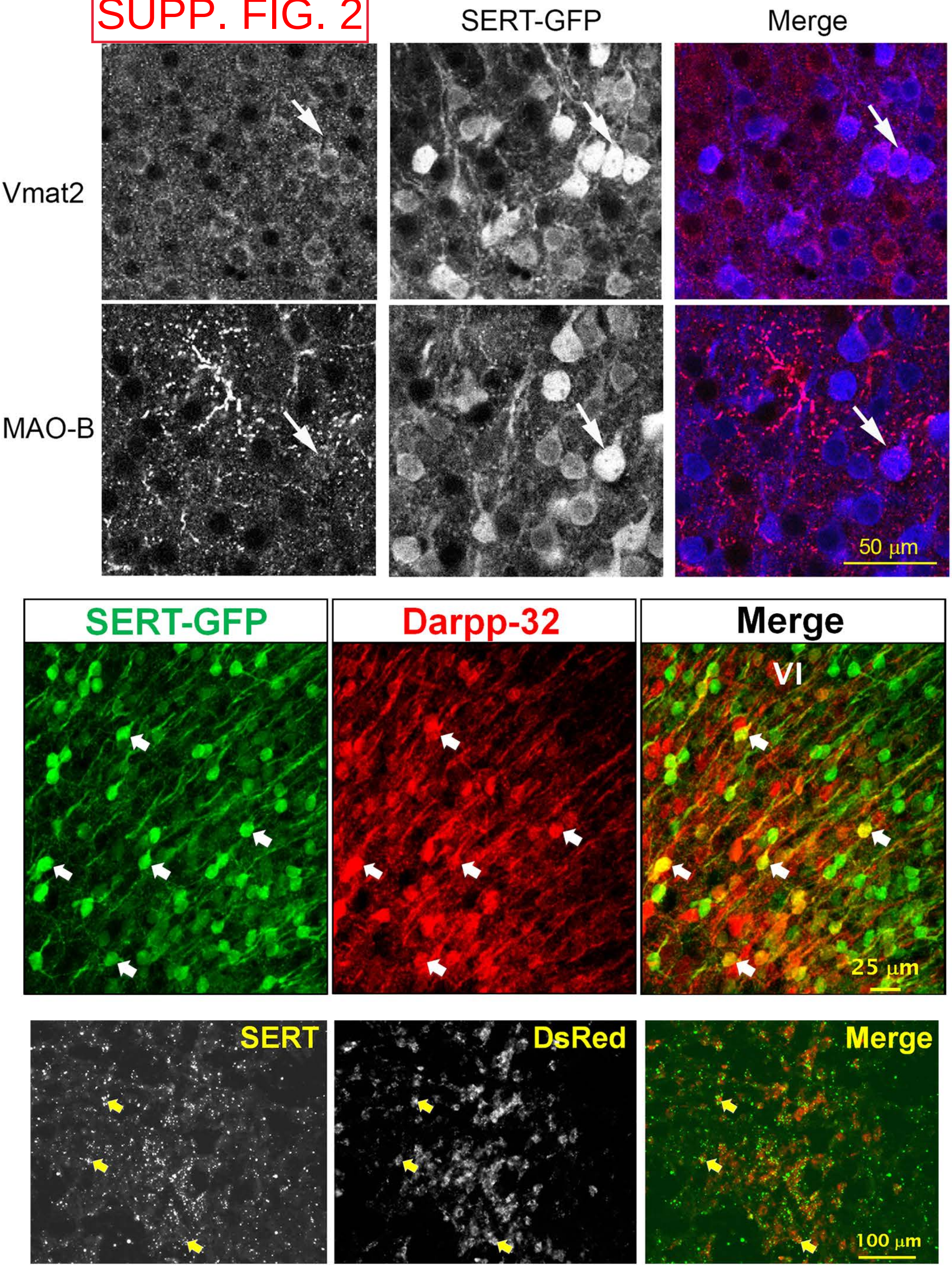
SERT-GFP
Merge
Vmat2
MAO-B
50 μm
SERT-GFP
Darpp-32
Merge
VI
25 μm
SERT
DsRed
Merge
100 μm

SUPP. FIG. 3

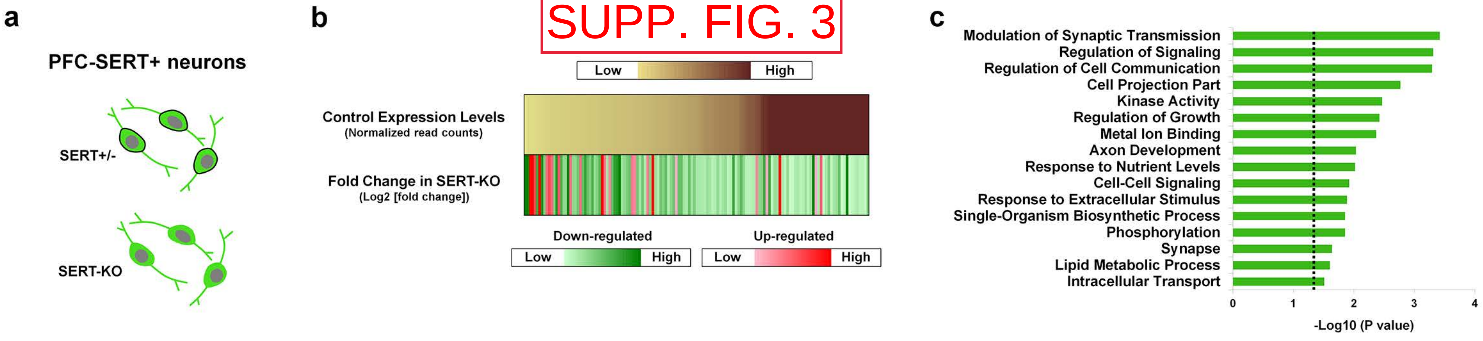
a
PFC-SERT+ neurons
SERT+/-
SERT-KO
b
Low
High
Control Expression Levels
(Normalized read counts)
Fold Change in SERT-KO
(Log2 [fold change])
Down-regulated
Low
High
Up-regulated
Low
High
c
Modulation of Synaptic Transmission
Regulation of Signaling
Regulation of Cell Communication
Cell Projection Part
Kinase Activity
Regulation of Growth
Metal Ion Binding
Axon Development
Response to Nutrient Levels
Cell-Cell Signaling
Response to Extracellular Stimulus
Single-Organism Biosynthetic Process
Phosphorylation
Synapse
Lipid Metabolic Process
Intracellular Transport
0
1
2
3
4
-Log10 (P value)

SUPP. FIG. 4

Structure	Axon Density
**Telencephalon**	
Agranular Insular Cortex	HIGH
Perirhinal Cortex	LOW
Piriform Cortex	HIGH
Retrosplenial Cortex	LOW
Accumbens N.	LOW
Amygdala (Basomedial)	LOW
Amygdala (Cortical)	LOW
Anterior Olfactory N.	VERY HIGH
Bed N. of Stria Terminalis	MEDIUM
Caudate Putamen	LOW
Claustrum	MEDIUM
Corpus Callosum	LOW
Diagonal Band N.	HIGH
Endopiriform N.	HIGH
Globus Pallidus	NONE
Hippocampus	NONE
Lateral Septum	LOW
Lateral Preoptic Area	HIGH
Medial Preoptic Area	HIGH
Medial Septal N.	HIGH
Olfactory Tubercle	HIGH
Ventral Pallidum	HIGH
**Diencephalon**	
Thalamus (Anterodorsal N.)	LOW
Thalamus (Anteromedial N.)	LOW
Thalamus (Anteroventral N.)	NONE
Thalamus (Central Medial N.)	VERY HIGH
Thalamus (Lat. Geniculate N.)	NONE
Thalamus (Lateral Habenula)	LOW
Thalamus (Laterodorsal N.)	MEDIUM
Thalamus (Lateroposterior N.)	LOW
Thalamus (Med. Gen. N.)	NONE
Thalamus (Medial Habenula)	NONE
Thalamus (Mediodorsal N.)	VERY HIGH
Thalamus (Parafascicular N.)	VERY HIGH
**Diencephalon (cont.)**	
Thalamus (Paratenial N.)	VERY HIGH
Thalamus (Paraventricular N.)	HIGH
Thalamus (Posterior N.)	VERY HIGH
Thalamus (Reticular N.)	MEDIUM
Thalamus (Reuniens N.)	VERY HIGH
Thalamus (Rhomboid N.)	VERY HIGH
Thalamus (Ventrobasal)	LOW
Hypothal. (Anterior N.)	MEDIUM
Hypothal. (Lateral N.)	VERY HIGH
Hypothal. (Paraventricular N.)	LOW
Hypothal. (Perifornical Area)	HIGH
Hypothal. (Posterior N.)	VERY HIGH
Hypothal. Supramammilary N.)	HIGH
Hypothal. (Ventromedial N.)	MEDIUM
Subthalamus	HIGH
**Brainstem**	
Dorsal Raphe N.	HIGH
Dorsal Tegmental N.	NONE
Interpeduncular N.	LOW
Laterodorsal Tegmental N.	HIGH
Locus Coeruleus	MEDIUM
Median Raphe N.	HIGH
Mesencephalic Ret. Formation	MEDIUM
Pontine Reticular N.	LOW
Parabrachial N.	HIGH
Periventricular Fiber System	HIGH
Pedunculopontine Tegment. N.	MEDIUM
Periaqueductal Gray	MEDIUM
Raphe Magnus N.	LOW
Retrorubral Area	VERY HIGH
Sust. Nigra Pars Compacta	VERY HIGH
Sust. Nigra Pars Reticulata	LOW
Supralemniscal N. (B9)	MEDIUM
Superior Colliculus	HIGH
Ventral Tegmental Area	VERY HIGH

NONE LOW MEDIUM HIGH VERY HIGH

SUPP. FIG. 5

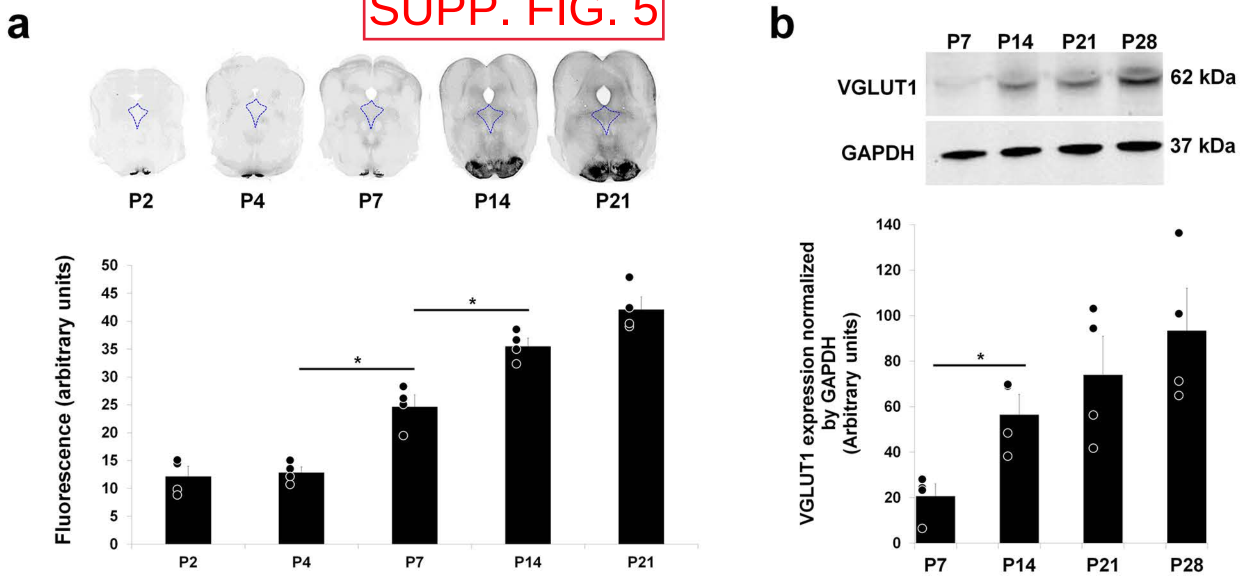
a
P2
P4
P7
P14
P21
Fluorescence (arbitrary units)
50
45
40
35
30
25
20
15
10
5
0
*
*
P2
P4
P7
P14
P21
b
P7
P14
P21
P28
VGLUT1
GAPDH
62 kDa
37 kDa
VGLUT1 expression normalized by GAPDH (Arbitrary units)
140
120
100
80
60
40
20
0
*
P7
P14
P21
P28

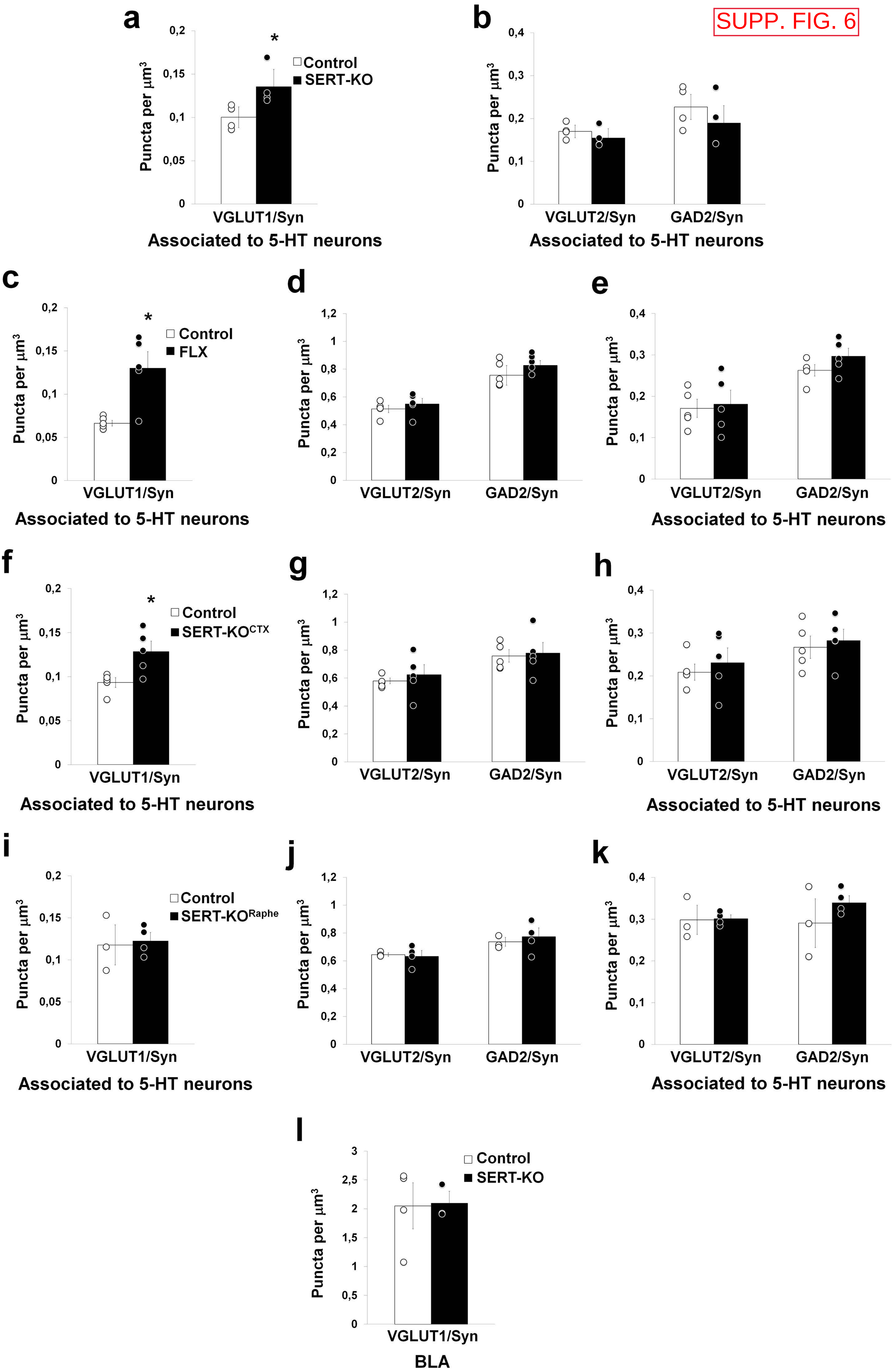

SUPP. FIG. 6
a
Puncta per µm³
0,2
0,15
0,1
0,05
0
*
Control
SERT-KO
VGLUT1/Syn
Associated to 5-HT neurons
b
0,4
0,3
0,2
0,1
0
VGLUT2/Syn
GAD2/Syn
Associated to 5-HT neurons
c
*
Control
FLX
VGLUT1/Syn
Associated to 5-HT neurons
d
1,2
1
0,8
0,6
0,4
0,2
0
VGLUT2/Syn
GAD2/Syn
e
VGLUT2/Syn
GAD2/Syn
Associated to 5-HT neurons
f
*
Control
SERT-KOCTX
VGLUT1/Syn
Associated to 5-HT neurons
g
VGLUT2/Syn
GAD2/Syn
h
VGLUT2/Syn
GAD2/Syn
Associated to 5-HT neurons
i
Control
SERT-KORaphe
VGLUT1/Syn
Associated to 5-HT neurons
j
VGLUT2/Syn
GAD2/Syn
k
VGLUT2/Syn
GAD2/Syn
Associated to 5-HT neurons
l
3
2,5
2
1,5
1
0,5
0
Control
SERT-KO
VGLUT1/Syn
BLA

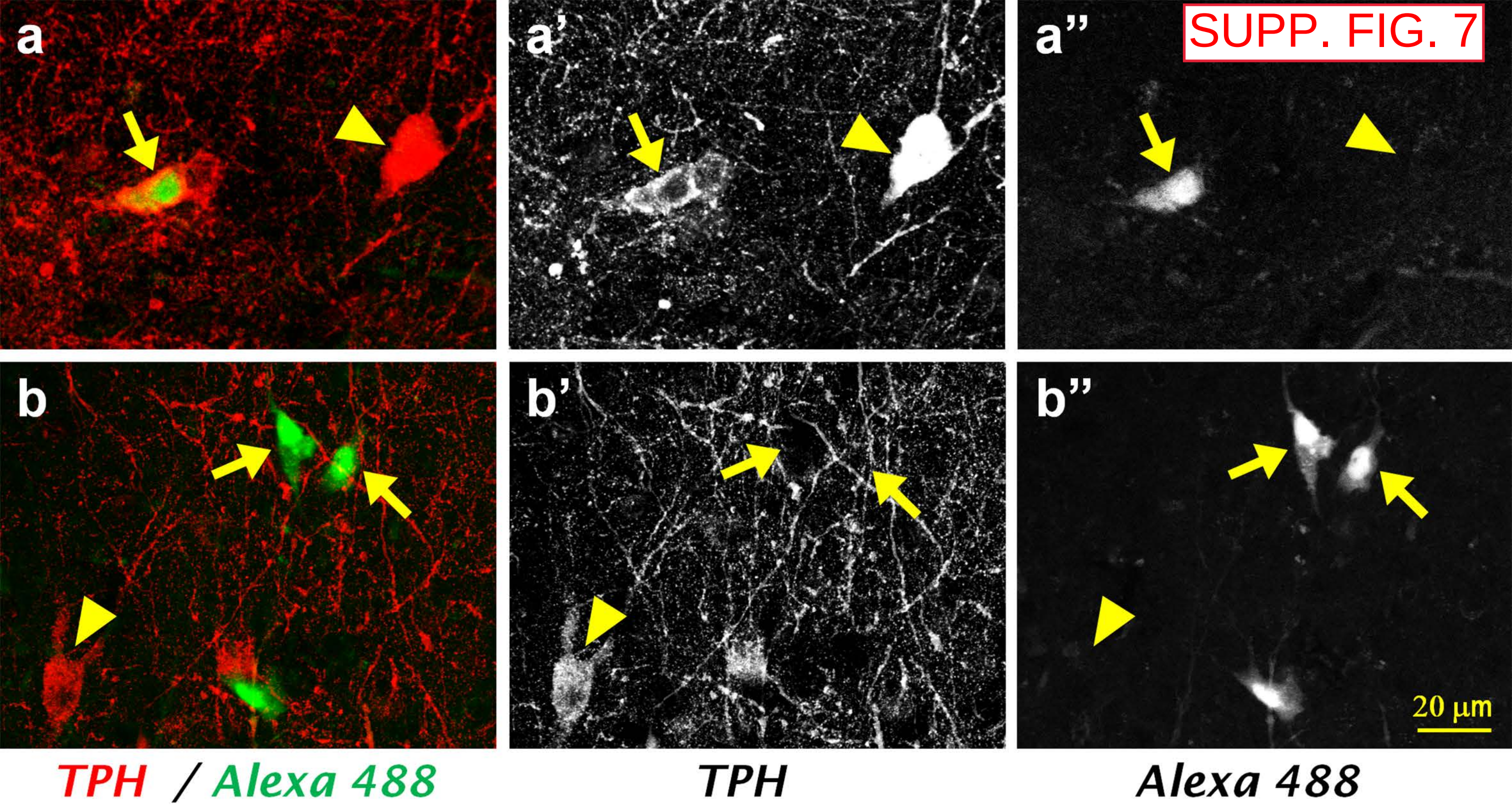
SUPP. FIG. 7
a
a'
a''
b
b'
b''
20 µm
TPH / Alexa 488
TPH
Alexa 488

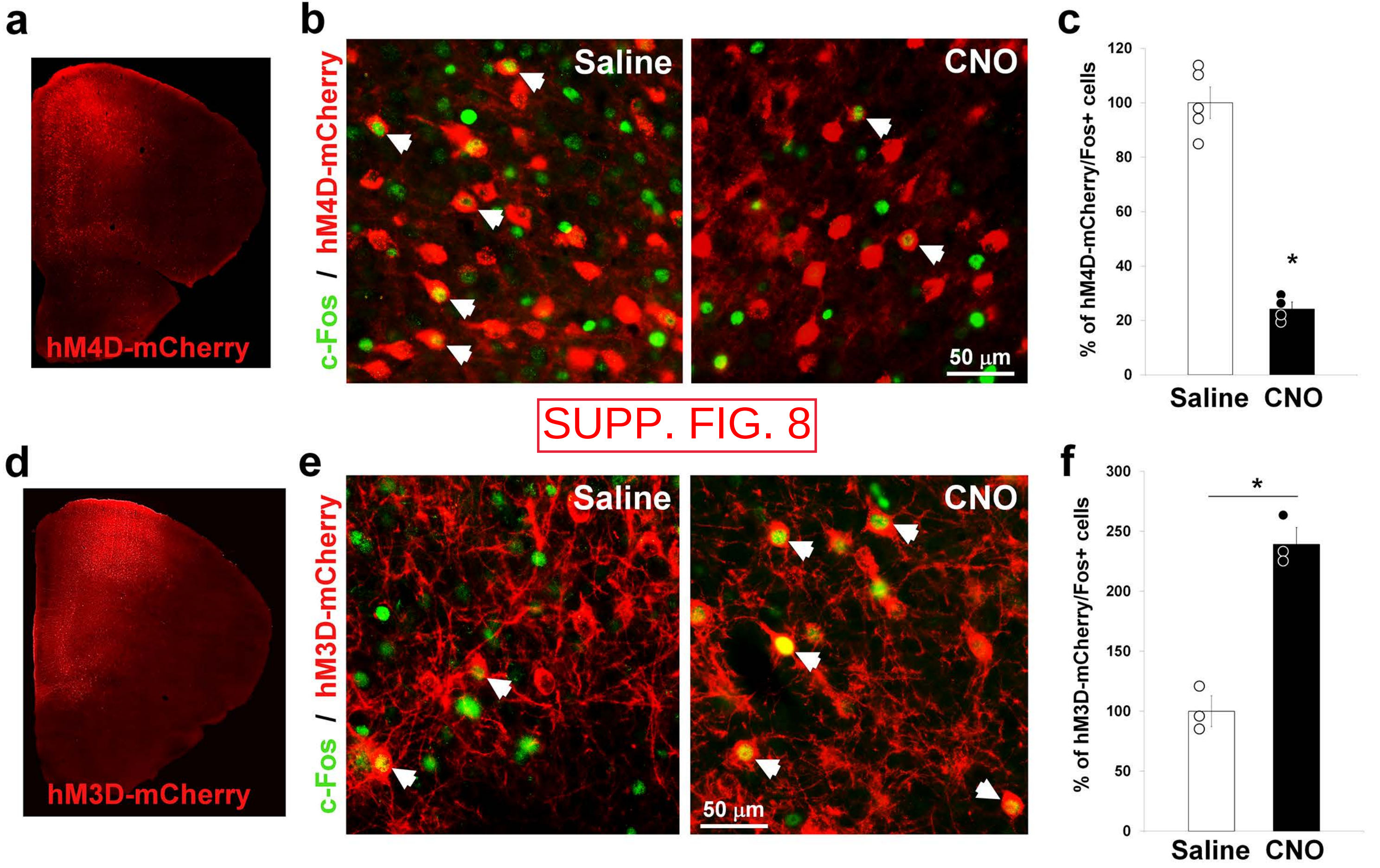
a
hM4D-mCherry
b
c-Fos / hM4D-mCherry
Saline
CNO
50 μm
c
% of hM4D-mCherry/Fos+ cells
120
100
80
60
40
20
0
*
Saline
CNO
SUPP. FIG. 8
d
hM3D-mCherry
e
c-Fos / hM3D-mCherry
Saline
CNO
50 μm
f
% of hM3D-mCherry/Fos+ cells
300
250
200
150
100
50
0
*
Saline
CNO

SUPP. FIG. 9

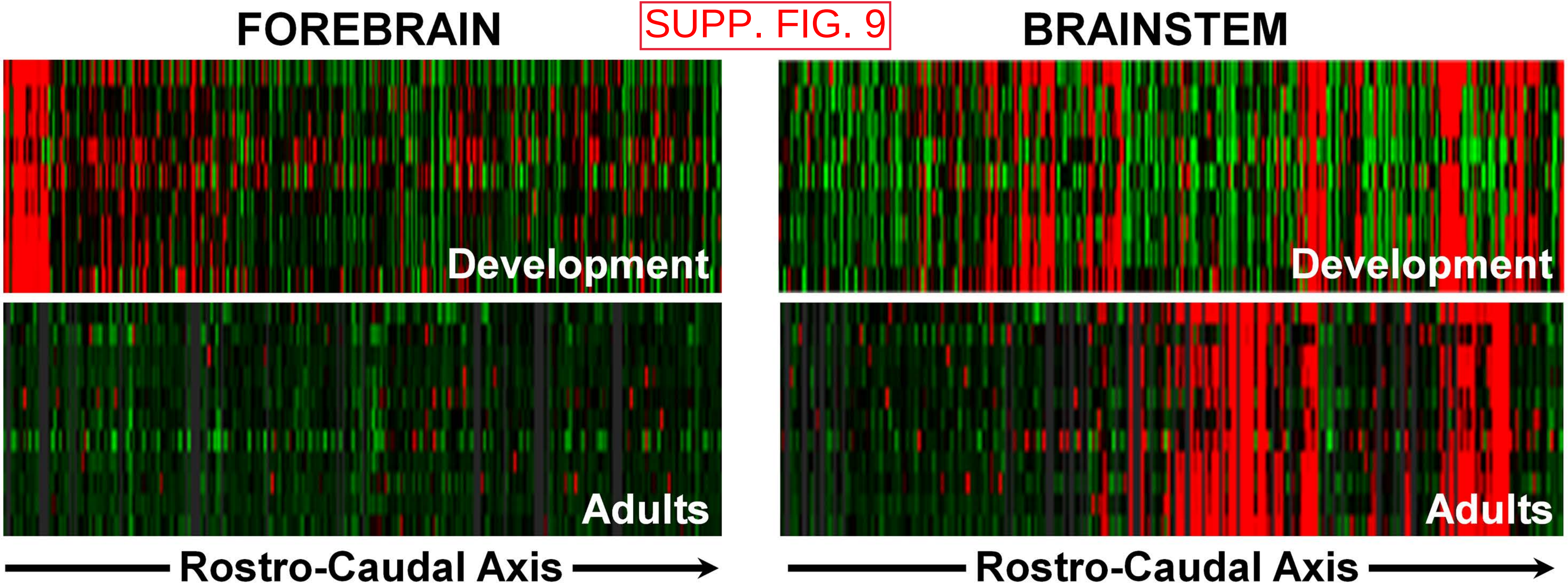
FOREBRAIN
BRAINSTEM
Development
Adults
Development
Adults
Rostro-Caudal Axis
Rostro-Caudal Axis

SUPP. TABLE 2

Gene	Log2 (Fold Change)	P value	Neuronal Function	References
Stmn1-rs1	-10,551	4,336E-42	Cytoskeleton Interaction/Neurite Growth/Synaptic Plasticity	[1–3]
Slc6a4	-4,044	3,440E-17	Serotonin Transport	[4]
Pcdhgc4	-1,623	2,703E-04	Cytoskeleton Interaction/Neurite Growth/Synaptogenesis	[5–7]
Flywch1	-1,616	1,347E-04	Unknown	-
Gna12	-1,586	2,024E-04	Neurite Growth/Synaptogenesis/Synaptic Plasticity	[8,9]
Telo2	-1,399	1,450E-03	Unknown	-
Pcdha12	-1,390	3,452E-03	Cytoskeleton Interaction/Axon Development	[10,11]
Clstn1	-1,365	6,130E-04	Cytoskeleton Interaction/Axon Branching/Synaptic Plasticity	[12,13]
Pla2g6	-1,360	4,187E-03	Neurite Growth/Axon, Synapse Remodeling	[14,15]
Qsox1	-1,360	4,093E-03	Extracellular Matrix Remodeling	[16]

Plcg1	-1,346	3,502E-03	Cytoskeleton Interaction/Neurite Growth	[17]
Gm1821	-1,316	2,691E-03	Unknown	-
Ppp1r37	-1,310	4,659E-03	Cytoskeleton Interaction/Neurite, Axon Growth/Synaptic Plasticity	[18–21]
Pcdha3	-1,302	7,749E-03	Cytoskeleton Interaction/Axon Development	[10,11]
Aldh3a1	-1,235	5,233E-03	Metabolism of Biogenic Amines/Axon, Synapse Maturation	[22,23]
Stk32c	-1,227	7,180E-03	Cytoskeleton Interaction/Neurite, Axon Growth	[24]
Pex10	-1,185	1,616E-02	Neurite, Axon Development	[25–27]
Pnpla3	-1,173	1,376E-02	Unknown	-
Lss	-1,141	7,224E-03	UNknown	-
Jun	-1,130	1,363E-02	Neurite, Axon Growth	[28–30]
Xkr4	-1,117	1,022E-02	Cytoskeleton Interaction/Axon Growth	[31]

Tmem150c	-1,092	1,069E-02	Unknown	-
Fads2	-1,087	8,003E-03	Differentiation	[32]
Slc20a2	-1,085	1,547E-02	Dendritic Cytoskeleton Interaction	[33]
Limk1	-1,054	1,281E-02	Cytoskeleton Interaction/Neurite, Axon Growth/Synapse Development and Plasticity	[34–37]
Ggt7	-1,049	8,041E-03	Glutamate Metabolism	[38,39]
Cdh18	-1,045	1,679E-02	Cytoskeleton Interaction/Neurite, Axon Growth/Synapse Development and Plasticity	[40–44]
Sh3bp5l	-1,038	1,157E-02	Cytoskeleton Interaction/ Axon Growth/Synapse Development	[45–47]
Clstn3	-1,024	9,889E-03	Synapse Development	[48,49]
Unk	-1,010	2,288E-02	Morphogenesis and Differentiation	[50,51]

References

1. Belmont, L. D. & Mitchison, T. J. Identification of a protein that interacts with tubulin dimers and increases the catastrophe rate of microtubules. *Cell* **84,** 623–631 (1996).
2. Shumyatsky, G. P. *et al.* stathmin, a gene enriched in the amygdala, controls both learned and innate fear. *Cell* **123,** 697–709 (2005).
3. Yamada, K. *et al.* Increased stathmin1 expression in the dentate gyrus of mice causes abnormal axonal arborizations. *PloS One* **5,** e8596 (2010).
4. Blakely, R. D. *et al.* Cloning and expression of a functional serotonin transporter from rat brain. *Nature* **354,** 66–70 (1991).
5. Weiner, J. A., Wang, X., Tapia, J. C. & Sanes, J. R. Gamma protocadherins are required for synaptic development in the spinal cord. *Proc. Natl. Acad. Sci. U. S. A.* **102,** 8–14 (2005).
6. Garrett, A. M. & Weiner, J. A. Control of CNS synapse development by {gamma}-protocadherin-mediated astrocyte-neuron contact. *J. Neurosci. Off. J. Soc. Neurosci.* **29,** 11723–11731 (2009).
7. Lefebvre, J. L., Kostadinov, D., Chen, W. V., Maniatis, T. & Sanes, J. R. Protocadherins mediate dendritic self-avoidance in the mammalian nervous system. *Nature* **488,** 517–521 (2012).
8. Kvachnina, E. *et al.* 5-HT7 receptor is coupled to G alpha subunits of heterotrimeric G12-protein to regulate gene transcription and neuronal morphology. *J. Neurosci. Off. J. Soc. Neurosci.* **25,** 7821–7830 (2005).
9. Kobe, F. *et al.* 5-HT7R/G12 signaling regulates neuronal morphology and function in an age-dependent manner. *J. Neurosci. Off. J. Soc. Neurosci.* **32,** 2915–2930 (2012).

10. Triana-Baltzer, G. B. & Blank, M. Cytoplasmic domain of protocadherin-alpha enhances homophilic interactions and recognizes cytoskeletal elements. *J. Neurobiol.* **66,** 393–407 (2006).

11. Hasegawa, S. *et al.* Constitutively expressed Protocadherin-α regulates the coalescence and elimination of homotypic olfactory axons through its cytoplasmic region. *Front. Mol. Neurosci.* **5,** 97 (2012).

12. Ponomareva, O. Y., Holmen, I. C., Sperry, A. J., Eliceiri, K. W. & Halloran, M. C. Calsyntenin-1 regulates axon branching and endosomal trafficking during sensory neuron development in vivo. *J. Neurosci. Off. J. Soc. Neurosci.* **34,** 9235–9248 (2014).

13. Ster, J. *et al.* Calsyntenin-1 regulates targeting of dendritic NMDA receptors and dendritic spine maturation in CA1 hippocampal pyramidal cells during postnatal development. *J. Neurosci. Off. J. Soc. Neurosci.* **34,** 8716–8727 (2014).

14. Forlenza, O. V., Mendes, C. T., Marie, S. K. N. & Gattaz, W. F. Inhibition of phospholipase A2 reduces neurite outgrowth and neuronal viability. *Prostaglandins Leukot. Essent. Fatty Acids* **76,** 47–55 (2007).

15. Sumi-Akamaru, H., Beck, G., Kato, S. & Mochizuki, H. Neuroaxonal dystrophy in PLA2G6 knockout mice. *Neuropathol. Off. J. Jpn. Soc. Neuropathol.* **35,** 289–302 (2015).

16. Mairet-Coello, G., Tury, A., Fellmann, D., Risold, P.-Y. & Griffond, B. Ontogenesis of the sulfhydryl oxidase QSOX expression in rat brain. *J. Comp. Neurol.* **484,** 403–417 (2005).

17. Itoh, K., Ishima, T., Kehler, J. & Hashimoto, K. Potentiation of NGF-induced neurite outgrowth in PC12 cells by papaverine: role played by PLC-γ, IP3 receptors. *Brain Res.* **1377,** 32–40 (2011).

18. Babu, K., Bahri, S., Alphey, L. & Chia, W. Bifocal and PP1 interaction regulates targeting of the R-cell growth cone in Drosophila. *Dev. Biol.* **288,** 372–386 (2005).

19. Monroe, J. D. & Heathcote, R. D. Protein phosphatases regulate the growth of developing neurites. *Int. J. Dev. Neurosci. Off. J. Int. Soc. Dev. Neurosci.* **31,** 250–257 (2013).

20. Siddoway, B. A. *et al.* An essential role for inhibitor-2 regulation of protein phosphatase-1 in synaptic scaling. *J. Neurosci. Off. J. Soc. Neurosci.* **33,** 11206–11211 (2013).

21. Lee, S., Pant, H. C. & Shea, T. B. Divergent and convergent roles for kinases and phosphatases in neurofilament dynamics. *J. Cell Sci.* **127,** 4064–4077 (2014).

22. Squires, L. N. *et al.* Serotonin catabolism and the formation and fate of 5-hydroxyindole thiazolidine carboxylic acid. *J. Biol. Chem.* **281,** 13463–13470 (2006).

23. Wishart, T. M. *et al.* Combining comparative proteomics and molecular genetics uncovers regulators of synaptic and axonal stability and degeneration in vivo. *PLoS Genet.* **8,** e1002936 (2012).

24. Hall, G. F. & Yao, J. Neuronal morphology, axonal integrity, and axonal regeneration in situ are regulated by cytoskeletal phosphorylation in identified lamprey central neurons. *Microsc. Res. Tech.* **48,** 32–46 (2000).

25. Faust, P. L. Abnormal cerebellar histogenesis in PEX2 Zellweger mice reflects multiple neuronal defects induced by peroxisome deficiency. *J. Comp. Neurol.* **461,** 394–413 (2003).

26. Krysko, O. *et al.* Neocortical and cerebellar developmental abnormalities in conditions of selective elimination of peroxisomes from brain or from liver. *J. Neurosci. Res.* **85,** 58–72 (2007).

27. Zhang, J. *et al.* A tuberous sclerosis complex signalling node at the peroxisome regulates mTORC1 and autophagy in response to ROS. *Nat. Cell Biol.* **15,** 1186–1196 (2013).

28. Hirai, S., Banba, Y., Satake, T. & Ohno, S. Axon formation in neocortical neurons depends on stage-specific regulation of microtubule stability by the dual leucine zipper kinase-c-Jun N-terminal kinase pathway. *J. Neurosci. Off. J. Soc. Neurosci.* **31,** 6468–6480 (2011).
29. Moore, D. L. & Goldberg, J. L. Multiple transcription factor families regulate axon growth and regeneration. *Dev. Neurobiol.* **71,** 1186–1211 (2011).
30. Lerch, J. K., Martínez-Ondaro, Y. R., Bixby, J. L. & Lemmon, V. P. cJun promotes CNS axon growth. *Mol. Cell. Neurosci.* **59,** 97–105 (2014).
31. Zhu, X. *et al.* Giant axon formation in mice lacking Kell, XK, or Kell and XK: animal models of McLeod neuroacanthocytosis syndrome. *Am. J. Pathol.* **184,** 800–807 (2014).
32. Simón, M. V. *et al.* Synthesis of docosahexaenoic acid from eicosapentaenoic acid in retina neurons protects photoreceptors from oxidative stress. *J. Neurochem.* **136,** 931–946 (2016).
33. Inden, M., Iriyama, M., Takagi, M., Kaneko, M. & Hozumi, I. Localization of type-III sodium-dependent phosphate transporter 2 in the mouse brain. *Brain Res.* **1531,** 75–83 (2013).
34. Meng, Y. *et al.* Abnormal spine morphology and enhanced LTP in LIMK-1 knockout mice. *Neuron* **35,** 121–133 (2002).
35. Eaton, B. A. & Davis, G. W. LIM Kinase1 controls synaptic stability downstream of the type II BMP receptor. *Neuron* **47,** 695–708 (2005).
36. Piccioli, Z. D. & Littleton, J. T. Retrograde BMP signaling modulates rapid activity-dependent synaptic growth via presynaptic LIM kinase regulation of cofilin. *J. Neurosci. Off. J. Soc. Neurosci.* **34,** 4371–4381 (2014).

37. Arastoo, M., Hacker, C., Popovics, P., Lucocq, J. M. & Stewart, A. J. Phospholipase C-η2 interacts with nuclear and cytoplasmic LIMK-1 during retinoic acid-stimulated neurite growth. *Histochem. Cell Biol.* **145,** 163–173 (2016).

38. Kvamme, E., Schousboe, A., Hertz, L., Torgner, I. A. & Svenneby, G. Developmental change of endogenous glutamate and gamma-glutamyl transferase in cultured cerebral cortical interneurons and cerebellar granule cells, and in mouse cerebral cortex and cerebellum in vivo. *Neurochem. Res.* **10,** 993–1008 (1985).

39. Jankásková, B., Lisý, V. & Stastný, F. Effect of gamma-glutamyl transpeptidase inhibitors on the transport of glutamate into neuronal and glial primary cultures. *Int. J. Dev. Neurosci. Off. J. Int. Soc. Dev. Neurosci.* **10,** 225–230 (1992).

40. Tang, L., Hung, C. P. & Schuman, E. M. A role for the cadherin family of cell adhesion molecules in hippocampal long-term potentiation. *Neuron* **20,** 1165–1175 (1998).

41. Bozdagi, O., Shan, W., Tanaka, H., Benson, D. L. & Huntley, G. W. Increasing numbers of synaptic puncta during late-phase LTP: N-cadherin is synthesized, recruited to synaptic sites, and required for potentiation. *Neuron* **28,** 245–259 (2000).

42. Bamji, S. X. *et al.* Role of beta-catenin in synaptic vesicle localization and presynaptic assembly. *Neuron* **40,** 719–731 (2003).

43. Bozdagi, O., Valcin, M., Poskanzer, K., Tanaka, H. & Benson, D. L. Temporally distinct demands for classic cadherins in synapse formation and maturation. *Mol. Cell. Neurosci.* **27,** 509–521 (2004).

44. Hayano, Y. *et al.* The role of T-cadherin in axonal pathway formation in neocortical circuits. *Dev. Camb. Engl.* **141,** 4784–4793 (2014).

45. Desai, C. J., Garrity, P. A., Keshishian, H., Zipursky, S. L. & Zinn, K. The Drosophila SH2-SH3 adapter protein Dock is expressed in embryonic axons and facilitates synapse formation by the RP3 motoneuron. *Dev. Camb. Engl.* **126,** 1527–1535 (1999).

46. Hing, H., Xiao, J., Harden, N., Lim, L. & Zipursky, S. L. Pak functions downstream of Dock to regulate photoreceptor axon guidance in Drosophila. *Cell* **97,** 853–863 (1999).

47. Antoine-Bertrand, J. *et al.* p120RasGAP Protein Mediates Netrin-1 Protein-induced Cortical Axon Outgrowth and Guidance. *J. Biol. Chem.* **291,** 4589–4602 (2016).

48. Pettem, K. L. *et al.* The specific α-neurexin interactor calsyntenin-3 promotes excitatory and inhibitory synapse development. *Neuron* **80,** 113–128 (2013).

49. Um, J. W. *et al.* Calsyntenins function as synaptogenic adhesion molecules in concert with neurexins. *Cell Rep.* **6,** 1096–1109 (2014).

50. Avet-Rochex, A. *et al.* Unkempt is negatively regulated by mTOR and uncouples neuronal differentiation from growth control. *PLoS Genet.* **10,** e1004624 (2014).

51. Murn, J. *et al.* Control of a neuronal morphology program by an RNA-binding zinc finger protein, Unkempt. *Genes Dev.* **29,** 501–512 (2015).

SUPP. TABLE 3

Gene	Log2 (Fold Change)	P value	Neuronal Function	References
Tuba1c	1,413	2,538E-03	Cytoskeleton/Neurite, Axon Growth/Synapse Maturation	[1–3]
Uty	1,319	1,744E-03	Chromatin Remodeling	[4,5]
Ddx3y	1,259	8,007E-04	Differentiation	[6,7]
Kdm5d	1,214	7,313E-03	Chromatin Remodeling	[6,8]
Sncaip	1,170	2,312E-02	Synaptic Function	[9–11]
Nedd9	1,134	2,232E-02	Neurite Growth	[12–14]
2610507I01Rik	1,092	3,403E-02	Unknown	-
Ubqln2	1,092	1,064E-02	Protein Metabolism/Dendritic, Synaptic Function	[15–18]
Map3k6	1,087	3,734E-02	Axon, Synapse Growth	[19–21]
Antxr1	1,084	3,724E-02	Cytoskeleton Interaction	[22]

G530011O06Rik	1,070	4,320E-02	Unknown	-
Plp1	1,058	2,849E-02	Neurite, Synapse Growth	[23,24]
3110047P20Rik	1,052	3,864E-02	Inflammasome/Axon Loss	[25]

References

1. Miller, F. D., Naus, C. C., Durand, M., Bloom, F. E. & Milner, R. J. Isotypes of alpha-tubulin are differentially regulated during neuronal maturation. *J. Cell Biol.* **105,** 3065–3073 (1987).
2. Caroni, P. & Becker, M. The downregulation of growth-associated proteins in motoneurons at the onset of synapse elimination is controlled by muscle activity and IGF1. *J. Neurosci. Off. J. Soc. Neurosci.* **12,** 3849–3861 (1992).
3. Paden, C. M. *et al.* Distribution of growth-associated class I alpha-tubulin and class II beta-tubulin mRNAs in adult rat brain. *J. Comp. Neurol.* **362,** 368–384 (1995).
4. Xu, J., Deng, X., Watkins, R. & Disteche, C. M. Sex-specific differences in expression of histone demethylases Utx and Uty in mouse brain and neurons. *J. Neurosci. Off. J. Soc. Neurosci.* **28,** 4521–4527 (2008).

5. Shpargel, K. B., Sengoku, T., Yokoyama, S. & Magnuson, T. UTX and UTY demonstrate histone demethylase-independent function in mouse embryonic development. *PLoS Genet.* **8,** e1002964 (2012).
6. Armoskus, C. *et al.* Identification of sexually dimorphic genes in the neonatal mouse cortex and hippocampus. *Brain Res.* **1562,** 23–38 (2014).
7. Vakilian, H. *et al.* DDX3Y, a Male-Specific Region of Y Chromosome Gene, May Modulate Neuronal Differentiation. *J. Proteome Res.* **14,** 3474–3483 (2015).
8. Xu, J., Deng, X. & Disteche, C. M. Sex-specific expression of the X-linked histone demethylase gene Jarid1c in brain. *PloS One* **3,** e2553 (2008).
9. Ribeiro, C. S., Carneiro, K., Ross, C. A., Menezes, J. R. L. & Engelender, S. Synphilin-1 is developmentally localized to synaptic terminals, and its association with synaptic vesicles is modulated by alpha-synuclein. *J. Biol. Chem.* **277,** 23927–23933 (2002).
10. Murray, I. J. *et al.* Synphilin in normal human brains and in synucleinopathies: studies with new antibodies. *Acta Neuropathol. (Berl.)* **105,** 177–184 (2003).
11. Krüger, R. The role of synphilin-1 in synaptic function and protein degradation. *Cell Tissue Res.* **318,** 195–199 (2004).
12. Bargon, S. D., Gunning, P. W. & O'Neill, G. M. The Cas family docking protein, HEF1, promotes the formation of neurite-like membrane extensions. *Biochim. Biophys. Acta* **1746,** 143–154 (2005).
13. Sasaki, T. *et al.* Nedd9 protein, a Cas-L homologue, is upregulated after transient global ischemia in rats: possible involvement of Nedd9 in the differentiation of neurons after ischemia. *Stroke J. Cereb. Circ.* **36,** 2457–2462 (2005).
14. Knutson, D. C. & Clagett-Dame, M. atRA Regulation of NEDD9, a gene involved in neurite outgrowth and cell adhesion. *Arch. Biochem. Biophys.* **477,** 163–174 (2008).

15. Deng, H.-X. *et al.* Mutations in UBQLN2 cause dominant X-linked juvenile and adult-onset ALS and ALS/dementia. *Nature* **477,** 211–215 (2011).

16. Gorrie, G. H. *et al.* Dendritic spinopathy in transgenic mice expressing ALS/dementia-linked mutant UBQLN2. *Proc. Natl. Acad. Sci. U. S. A.* **111,** 14524–14529 (2014).

17. Zhang, K. Y., Yang, S., Warraich, S. T. & Blair, I. P. Ubiquilin 2: a component of the ubiquitin-proteasome system with an emerging role in neurodegeneration. *Int. J. Biochem. Cell Biol.* **50,** 123–126 (2014).

18. Radzicki, D., Liu, E., Deng, H.-X., Siddique, T. & Martina, M. Early Impairment of Synaptic and Intrinsic Excitability in Mice Expressing ALS/Dementia-Linked Mutant UBQLN2. *Front. Cell. Neurosci.* **10,** 216 (2016).

19. Hammarlund, M., Nix, P., Hauth, L., Jorgensen, E. M. & Bastiani, M. Axon regeneration requires a conserved MAP kinase pathway. *Science* **323,** 802–806 (2009).

20. Yan, D., Wu, Z., Chisholm, A. D. & Jin, Y. The DLK-1 kinase promotes mRNA stability and local translation in C. elegans synapses and axon regeneration. *Cell* **138,** 1005–1018 (2009).

21. Brace, E. J., Wu, C., Valakh, V. & DiAntonio, A. SkpA restrains synaptic terminal growth during development and promotes axonal degeneration following injury. *J. Neurosci. Off. J. Soc. Neurosci.* **34,** 8398–8410 (2014).

22. Garlick, K. M. & Mogridge, J. Direct interaction between anthrax toxin receptor 1 and the actin cytoskeleton. *Biochemistry (Mosc.)* **48,** 10577–10581 (2009).

23. Alfonso, J., Fernández, M. E., Cooper, B., Flugge, G. & Frasch, A. C. The stress-regulated protein M6a is a key modulator for neurite outgrowth and filopodium/spine formation. *Proc. Natl. Acad. Sci. U. S. A.* **102,** 17196–17201 (2005).

24. Huang, K.-Y. *et al.* Phosphorylation of the zebrafish M6Ab at serine 263 contributes to filopodium formation in PC12 cells and neurite outgrowth in zebrafish embryos. *PloS One* **6,** e26461 (2011).

25. Soulika, A. M. *et al.* Initiation and progression of axonopathy in experimental autoimmune encephalomyelitis. *J. Neurosci. Off. J. Soc. Neurosci.* **29,** 14965–14979 (2009).

Supplementary Figure 1. 5-HT reuptake by PFC-SERT+ neurons. After *in vivo* blockade of 5-HT degradation with the monoamine oxidase A inhibitor, clorgyline **(b)**, accumulation of 5-HT is visible in the cell bodies of cortical pyramidal neurons (arrows) in clorgyline **c**) but not in saline treated mice **(a)**. **(d-e)** *In vivo* clorgyline treatment in SERT-TdTomato mice revealed the selective accumulation of 5-HT in PFC SERT-Tdtomato neurons (arrows in **e**). This is only evident when 5-HT degradation is blocked by clorgyline **(e)** but not in the saline-treated mice **(d)** or after clorgyline + fluoxetine (FLX) treatment **(f)**. Arrowheads indicate typical 5-HT raphe axons in the PFC, that are unaffected by the treatments.

Supplementary Figure 2. Validation of monoamine-related genes and DARPP-32 in SERT-GFP mice. Immunohistochemistry of the vesicular monoamine transporter type 2 (Vmat2, upper panels) and the enzyme monoamine oxidase B (MAO-B, upper panels), and DARPP-32 (middle panel) in PFC-SERT$^{Cre/+}$ neurons expressing GFP (SERT$^{Cre/+}$::RCE-EGFP). Fluorescent *in situ* hybridization of SERT and SERT-DsRed (lower panel) in the SERT$^{Cre/+}$::TdTomato mouse. Arrows indicate instances of double labeling. Antibodies used: rabbit antiserum anti-Vmat2 (1/1000, H-V004, Phoenix Pharmaceuticals Inc., Burlingame, USA), rabbit antiserum anti-MAO-B (1/1000, from Vitalis et al., 2003 [doi.org/10.1002/cne.10804]), and a rabbit monoclonal antibody anti-DARPP-32 (1/1000, #2306S, Cell Signaling Technologies, France).

Supplementary Figure 3. SERT invalidation alters developmental gene networks in PFC-SERT+ neurons. **(a)** EGFP-expressing neurons in the PFC were isolated from Sert $^{+/-}$ and Sert $^{-/-}$ mice (SERT$^{Cre/+}$::RCE-EGFP and SERT$^{Cre/Cre}$::RCE-EGFP,

respectively). The RNAs obtained from these cells were used for transcriptome profiling after deep sequencing. **(b)** Heatmaps of control gene expression levels and fold changes in differential gene expression when SERT is invalidated. The upper map shows the normalized read counts (from low to high) in control PFC neurons ($SERT^{Cre/+}$::RCE-EGFP) of genes significantly changed in the subsequent differential expression analysis. The heatmap below shows fold-changes of differentially-expressed genes (down-regulated or upregulated) in PFC neurons in $SERT^{-/-}$ mice ($SERT^{Cre/Cre}$::RCE-EGFP). **(c)** Top altered gene networks obtained with gene ontology analysis of differentially-expressed genes in $SERT^{-/-}$ mice. Enrichment threshold was set at 1.5 with p<0.05 (indicated by dashed line).

Supplementary Figure 4. Neuroanatomical targets of PFC-SERT+ neurons. Main brain regions targeted by PFC-SERT+ neuron axons as revealed by conditional anterograde viral tracing. After injection of AAV2/1-CAG-LSL-EGFP-bGH in the PFC of $SERT^{Cre/+}$ mice at P4-P5 (n = 15), a heat map was made using a subjective quantitative color-coded score for axon density within different brain regions. Analyzed regions were selected based on previous tract-tracing studies describing the main neuroanatomical targets of PFC projection-neurons.

Supplementary Figure 5. Maturation of cortical axon projections to their subcortical targets. **(a)** Postnatal ontogeny of cortical descending axon-projections in the DRN using the $EMX1b^{Cre/+}$::Tdtomato mouse. We quantified mean values of red fluorescence at the targets (delineated by blue lines) at different ages (4 mice/age). $F_{4,15} = 70.79$, $p<10^{-8}$; P4 vs. P7, and P7 vs. P14, *p<0.001; P2 vs. P4, p=0.99, and P14 vs.

P21, p=0.07. Tukey's test after one-way ANOVA. **(b)** Ontogenetic analysis of VGLUT1 expression levels in the DRN during postnatal development assessed by western blot. Upper panel: representative western blots of VGLUT1 and GAPDH expression in the DRN. Lower panel: quantitative analysis of VGLUT1 expression levels normalized by GAPDH expression (Welch's statistic = 9.85, *p<0.01; P7 vs. P14, *p<0.05; P14 vs. P21, p=0.73, and P21 vs. P28, p=0.82. Games-Howell post-hoc test. Error bars represent S.E.M.

Supplementary Figure 6. Array tomography quantitative analysis of glutamate and GABAergic synaptic afferents to the DRN, and their associations to 5-HT neurons. **(a-b)** Lack of SERT increases the density of cortical synaptic boutons (VGLUT1+) associated with 5-HT cells **(a)** (4 mice/genotype; $F_{1,6}$ = 6.63, *p<0.05), without changing the number of VGLUT2+ or GAD2+ axon boutons associated with 5-HT cells **(b)** ($F_{1,6}$ = 1.13, p=0.33 and $F_{1,6}$ = 0.91, p=0.38, respectively). **(c-e)** Pharmacological SERT blockade by fluoxetine increases the density of cortical synaptic boutons (VGLUT1+) associated with 5-HT cells **(c)** (5 mice/genotype; $F_{1,8}$ = 13.25, *p<0.01), without changing the number of VGLUT2+ or GAD2+ axon boutons **(d)** ($F_{1,8}$ = 0.67, p=0.44 and $F_{1,8}$ = 1.04, p=0.34, respectively) or their associations with 5-HT cells **(e)** ($F_{1,8}$ = 0.08, p=0.79 and $F_{1,8}$ = 2.38, p=0.16, respectively). **(f-h)** Conditional deletion of cortical SERT (SERT-KO^{CTX}) increases the density of cortical synaptic boutons (VGLUT1+) associated with 5-HT cells **(f)** (5 mice/genotype; $F_{1,8}$ = 8.69, *p<0.02), without changing either the number of VGLUT2+ or GAD2+ axon boutons **(g)** ($F_{1,8}$ = 0.44, p=0.53 and $F_{1,8}$ = 0.06, p=0.81, respectively) or their associations with 5-HT cells **(h)** ($F_{1,8}$ = 0.39, p=0.55 and $F_{1,8}$ = 0.22, p=0.65, respectively). **(i-k)** Conditional deletion of SERT from raphe neurons

(SERT-KO^{Raphe}) does not modify the density of cortical synaptic boutons (VGLUT1+) associated with 5-HT cells **(i)** (3-4 mice/genotype; $F_{1,5} = 0.06$, p=0.81), or the number of VGLUT2+ and GAD2+ axon boutons **(j)** ($F_{1,5} = 0.07$, p=0.80 and $F_{1,5} = 0.29$, p=0.61, respectively) nor their association with 5-HT cells **(k)** ($F_{1,5} = 0.02$, p=0.91 and $F_{1,5} = 1.27$, p=0.31, respectively). **(l)** Lack of SERT does not change the density of VGLUT1+ synaptic boutons in the basolateral nucleus of the amygdala (BLA) (3-4 mice/genotype; $T_5 = 0.1042$, p=0.92). Data analyzed by one-way ANOVA **(a-k)** and t-test **(l)**. Error bars represent S.E.M.

Supplementary Figure 7. Post-hoc identification of the two types of neurons recorded from the dorsal raphe in the *ex-vivo* electrophysiological experiments. After electroporation with Alexa 488 using the patch pipette, 5-HT **(a-a")** and non-5-HT **(b-b")** neurons were identified by immunolabeling against TPH. Arrows indicate 5-HT positive neurons, while arrowheads points at recorded cells containing Alexa 488.

Supplementary Figure 8. Pharmacogenetic manipulation of PFC glutamate-projection neuron's activity. AAV5-CaMKIIa-hM4D(Gi)-mCherry or AAV8-CaMKIIa-hM3D(Gq)-mCherry was efficiently transduced in pyramidal neurons of the prelimbic, infralimbic and orbital regions **(a-b** and **d-e)**. In hM4D mice, PFC activation elicited by acute swim stress was robustly decreased by about 80% by the acute pre-treatment with CNO (1mg/kg) administered 30 min before the swim **(b,c)** (5-4 mice/treatment; $T_7 = 12.03$, $p<10^{-5}$). Conversely, in hM3D mice, CNO treatment elicits a large increase in the activation of PFC glutamate neurons, evidenced by an increase in c-Fos expression levels **(e-f)** (3 mice/treatment; $T_4 = 8.892$, p<0.001). Immunohistochemistry for c-Fos in

mCherry-expressing neurons was used as readout of neuronal activity. The chicken antibody anti-mCherry (1:1000, AB205402, Abcam, France) and rabbit anti-c-Fos antiserum (1:1000, AB190289, Abcam, France) were used. Data were analyzed by t-test. Error bars represent S.E.M.

Supplementary Figure 9. Fetal and adult SERT expression in humans. Transcriptional data obtained from Brainspan Atlas of the Developing Human Brain (http://www.brainspan.org). **(a)** In the fetal human brain SERT expression is present in both fronto-cortical regions and brainstem structures. **(b)** In the adult brain, SERT is mostly expressed in brainstem regions.

Supplementary Table 1. Genes differentially expressed after SERT invalidation in PFC-SERT+ neurons at P7. All genes with $p<0.05$.

Supplementary Table 2. Top genes down-regulated by SERT invalidation in PFC-SERT+ neurons at P7. A threshold of 100 reads for normalized expression levels was set with a $p<0.05$. Differential expression is shown as Log2(fold change). The reported roles of differentially-expressed genes in different aspects of neuronal development are indicated together with their supporting references.

Supplementary Table 3. Top genes up-regulated by SERT invalidation in PFC-SERT+ neurons at P7. A threshold of 100 reads for normalized expression levels was set with a $p<0.05$. Differential expression is shown as Log2(fold change). The reported

roles of differentially-expressed genes in different aspects of neuronal development are indicated together with their supporting references.

Supplementary Table 4. Gene ontology of differentially-expressed genes after SERT invalidation in PFC-SERT+ neurons at P7 using DAVID Bioinformatics Resources, NIAID/NIH.
