# Supplementary material for: SSRIs target prefrontal to raphe circuits during development modulating synaptic connectivity and emotional behavior
Source: Mol Psychiatry. 2018 Oct 2;24(5):726–45. doi: 10.1038/s41380-018-0260-9 (PMC6445781; doi:10.1038/s41380-018-0260-9)
Supplement: Supplementary file 2 — Supplementary table 1 [file 41380_2018_260_MOESM2_ESM.pdf]

Gene	Mean Reads	Reads Ctrol	Reads KO	Fold Change	log2 FC	Reads (Ctrol-KO)	P value
Stmn1-rs1	507,58017	1014,48417	0,67617263	0,00066652	-10,5510671	1013,807995	4,3363E-42
Slc6a4	943,844123	1779,77653	107,911714	0,06063217	-4,04377272	1671,864818	3,4404E-17
Pcdhga9	7,67415607	14,0178049	1,33050722	0,09491552	-3,3972122	12,68729769	0,00727627
Rims4	16,1159609	26,4804101	5,75151172	0,21719874	-2,20291236	20,72889836	0,00686227
Fcrls	59,099385	94,8568999	23,3418702	0,24607456	-2,02283256	71,51502965	0,00024234
Pold1	40,9196398	65,3574901	16,4817895	0,25217905	-1,98747969	48,87570059	0,00077209
Pcdhgb6	25,0621162	39,085147	11,0390854	0,28243684	-1,82399981	28,04606154	0,00707109
Pcdhga7	37,9747573	59,113732	16,8357827	0,28480324	-1,81196251	42,27794935	0,00324623
Laptm5	60,5714076	93,7960515	27,3467638	0,2915556	-1,77815708	66,44928776	0,00093988
Ccne1	59,798783	91,4808059	28,11676	0,30735147	-1,70203872	63,36404596	0,0029225
Pcdhb8	47,9991728	73,0815376	22,9168079	0,31357862	-1,67310088	50,16472967	0,00483444
Slit3	23,5746476	35,645517	11,5037782	0,32272721	-1,63161288	24,14173881	0,01855693
Pcdhgc4	165,329652	249,595745	81,0635591	0,32477941	-1,62246792	168,5321862	0,00027026
Flywch1	307,388929	463,57567	151,202187	0,32616506	-1,61632584	312,3734826	0,00013469
Gna12	289,853981	434,857648	144,850314	0,33309823	-1,5859804	290,0073344	0,0002024
Lama2	51,8626249	76,953773	26,7714768	0,34789037	-1,52329534	50,18229622	0,01131334
Pcdha10	42,1369322	62,3550312	21,9188332	0,35151667	-1,50833497	40,43619807	0,01023787
2210408F21I	53,8647869	79,102666	28,6269078	0,36189561	-1,46635448	50,47575822	0,01945091
Telo2	202,169455	293,194842	111,144068	0,37907921	-1,39942877	182,0507736	0,00144999
Gpam	61,9550633	89,6994547	34,2106719	0,38139219	-1,39065278	55,48878281	0,0084675
Pcdha12	91,4743701	132,422573	50,5261671	0,38155252	-1,39004642	81,89640596	0,00345213
Snx8	50,0715918	72,3247729	27,8184108	0,38463184	-1,37844989	44,50636218	0,01158036
Myo7a	50,8599768	73,4322631	28,2876906	0,38522155	-1,37623966	45,14457254	0,01983551
Clstn1	917,830447	1322,18675	513,474141	0,38835221	-1,36456244	808,7126115	0,00061303
Pla2g6	103,806564	149,405178	58,2079501	0,38959794	-1,35994204	91,1972283	0,00418726
Qsox1	108,151316	155,658247	60,6443847	0,38959956	-1,35993604	95,01386195	0,00409302
Ano8	67,6501738	97,1691715	38,131176	0,39242051	-1,34952764	59,03799549	0,00649498
Plcg1	115,618927	165,949018	65,288837	0,39342708	-1,34583184	100,6601807	0,00350157
Fam171a2	76,1606275	109,039735	43,2815196	0,39693346	-1,33303091	65,75821579	0,00556217
Gm1821	19933,9907	28443,8883	11424,093	0,40163612	-1,31603906	17019,79526	0,00269114
Zfp768	48,1875451	68,7250738	27,6500163	0,40232792	-1,31355622	41,07505751	0,01466565
Ppp1r37	101,352641	144,459317	58,2459657	0,40319979	-1,31043322	86,21335144	0,00465868
Pcdha3	90,6896306	129,034655	52,3446067	0,40566317	-1,30164578	76,69004781	0,00774867

Pcdha2	75,0362642	106,5097	43,5628287	0,40900339	-1,28981528	62,94687098	0,01065707
Heatr2	68,9585575	96,9703847	40,9467302	0,42226016	-1,24379596	56,02365441	0,01399259
Pcdha11	82,3524129	115,651679	49,0531466	0,42414556	-1,23736862	66,59853252	0,0088682
Aldh3a1	857,039188	1202,96549	511,112882	0,42487743	-1,2348814	691,8526111	0,00523316
Aaed1	41,6624565	58,4435919	24,8813212	0,42573224	-1,23198175	33,56227069	0,04884464
Stk32c	123,16918	172,589111	73,7492489	0,42731114	-1,22664118	98,83986161	0,00717973
Rhpn1	33,7177331	47,2103542	20,2251119	0,42840416	-1,22295562	26,98524223	0,03547139
Pcdha7	54,8313922	76,7440135	32,9187709	0,42894252	-1,22114375	43,82524262	0,02403679
Taf1c	56,236087	78,4592328	34,0129413	0,43351101	-1,20585946	44,4462915	0,0235997
Pex10	93,5344928	129,920209	57,1487764	0,43987596	-1,18483135	72,7714328	0,01616354
Pnpla3	185,842069	257,490004	114,194133	0,44348958	-1,17302789	143,2958704	0,0137546
4931428F04I	300,871261	414,965024	186,777498	0,4501042	-1,15166908	228,1875254	0,00493865
Lss	269,180257	370,429203	167,931311	0,45334253	-1,14132659	202,4978923	0,00722415
Jun	103,517579	142,117351	64,9178061	0,45679015	-1,13039655	77,19954474	0,01363008
Mapk15	47,0258469	64,4838593	29,5678346	0,45853078	-1,1249095	34,91602469	0,04508129
Samd5	49,6130848	67,9792818	31,2468877	0,4596531	-1,12138262	36,73239413	0,04797393
Xkr4	218,218374	298,688844	137,747905	0,46117526	-1,11661297	160,9409385	0,0102209
Cpne7	48,5302911	66,4194474	30,6411349	0,46132776	-1,11613598	35,77831244	0,04712262
Xylt1	46,5980112	63,6479259	29,5480966	0,46424288	-1,1070483	34,09982923	0,03989366
Mlc1	57,1386324	77,8718368	36,4054279	0,46750442	-1,09694809	41,46640894	0,03969767
Polr3g	71,1964927	96,9598445	45,433141	0,46857688	-1,09364234	51,52670349	0,04796489
Tmem150c	178,781925	243,360664	114,203185	0,46927545	-1,0914931	129,1574787	0,01069152
Fads2	438,505031	596,339197	280,670866	0,47065641	-1,08725385	315,6683307	0,00800253
Slc20a2	170,463913	231,681993	109,245833	0,47153355	-1,08456767	122,43616	0,01546913
Pcdha5	91,5069663	124,044935	58,9689974	0,47538416	-1,07283426	65,07593767	0,02620703
1500009C09I	619,375263	837,581432	401,169095	0,4789613	-1,06201899	436,4123373	0,01708532
Limk1	158,612868	214,109733	103,116003	0,48160353	-1,05408213	110,99373	0,01280883
Ggt7	968,094754	1305,23032	630,959191	0,48340832	-1,0486858	674,2711263	0,00804082
Ralgps1	71,5649082	96,4422486	46,6875679	0,48409871	-1,04662685	49,7546807	0,03378437
Cdh18	388,064525	522,76143	253,367621	0,48467161	-1,04492053	269,3938084	0,01679024
Sh3bp5l	396,154433	532,815476	259,493391	0,487023	-1,03793819	273,3220844	0,01156963
Clstn3	748,351112	1003,21805	493,484175	0,49190121	-1,02355948	509,7338723	0,00988872
Unk	126,851571	169,54208	84,1610622	0,4964022	-1,01041858	85,38101827	0,0228839
Lrrc47	79,7694656	106,519931	53,0190003	0,49773784	-1,00654202	53,50093057	0,03736322

Cbs	117,078509	155,893105	78,2639129	0,50203576	-0,99413798	77,62919223	0,04354626
Aldh2	180,326137	240,055965	120,596309	0,50236747	-0,99318503	119,4596563	0,0181119
BC005764	951,340931	1266,3502	636,331667	0,50249265	-0,9928256	630,0185287	0,00886561
Lzts2	65,5815679	87,1130215	44,0501142	0,50566624	-0,98374263	43,06290735	0,04353275
Wwc1	162,388992	215,531725	109,24626	0,50686858	-0,98031635	106,2854648	0,02599855
Synpr	138,930669	183,5188	94,3425369	0,5140756	-0,95994757	89,17626347	0,04830051
Med25	531,61235	701,940473	361,284227	0,51469354	-0,95821442	340,6562468	0,01377957
Pfkl	873,500568	1151,28272	595,718414	0,51743886	-0,9505397	555,5643084	0,01631295
Ttll11	83,3593364	109,72697	56,9917029	0,51939558	-0,94509436	52,73526699	0,04741467
Tle2	121,939159	160,362097	83,5162204	0,52079776	-0,94120486	76,84587659	0,02832199
Chst15	134,503075	176,784504	92,2216461	0,52166137	-0,9388145	84,56285823	0,03959508
St3gal3	147,669922	194,036834	101,303011	0,52208134	-0,93765349	92,7338231	0,02918148
Cadm4	516,068803	678,065958	354,071648	0,52217877	-0,93738429	323,9943102	0,01650919
St6galnac6	423,444424	556,19661	290,692239	0,52264295	-0,9361024	265,5043715	0,01936418
Wdr6	1790,85357	2348,58043	1233,1267	0,52505194	-0,92946796	1115,453729	0,01692312
Pcdhga11	102,092921	133,841938	70,3439038	0,52557445	-0,92803294	63,49803455	0,04861483
Swsap1	158,869419	207,966467	109,772372	0,52783688	-0,92183593	98,19409479	0,04435179
Gpr27	260,061636	339,916411	180,206862	0,53015052	-0,91552607	159,7095485	0,0234788
Sema6b	176,639034	230,552951	122,725117	0,53230773	-0,90966759	107,8278338	0,02817257
Mafk	98,8645271	128,983972	68,745082	0,53297383	-0,90786339	60,23889029	0,0439298
Ppp2r5d	849,690354	1108,31851	591,062202	0,53329634	-0,90699067	517,2563028	0,01933426
Dgkh	197,078049	256,913484	137,242614	0,53419779	-0,9045541	119,6708698	0,03730719
Pkn1	184,036387	239,723179	128,349595	0,53540753	-0,90129067	111,373584	0,03122319
Zbtb17	191,170286	248,608231	133,73234	0,53792402	-0,89452567	114,8758912	0,03676392
Mib2	260,967361	339,025387	182,909335	0,53951516	-0,8902646	156,1160519	0,02981248
Agap3	1767,36362	2288,08264	1246,64461	0,54484248	-0,87608891	1041,438027	0,01986145
Aldh1b1	590,482529	763,722154	417,242904	0,54632814	-0,87216036	346,4792503	0,02616353
Fam109a	169,505474	218,852539	120,158409	0,54903822	-0,8650215	98,69412949	0,03373375
Zgpat	251,783198	324,287897	179,278499	0,55283746	-0,85507271	145,0093986	0,04202595
D10Jhu81e	260,58025	335,597106	185,563393	0,55293502	-0,85481814	150,0337132	0,03996914
Psap	4276,33351	5501,6607	3051,00632	0,55456097	-0,850582	2450,65438	0,0250997
Scyl1	555,488247	714,635352	396,341142	0,55460612	-0,85046455	318,2942101	0,02791868
Akt1	2108,31954	2709,11903	1507,52005	0,55646136	-0,8456466	1201,598984	0,02325585
Map3k12	441,789441	566,45838	317,120502	0,55983019	-0,83693881	249,3378782	0,0352414

Dpp3	1233,51937	1580,72034	886,318395	0,56070538	-0,83468519	694,4019494	0,03520026
Lrrc45	193,792725	248,006324	139,579126	0,5628047	-0,82929371	108,4271983	0,04086624
Csnk1g2	739,879212	945,057106	534,701318	0,56578731	-0,82166828	410,3557886	0,03282648
Podxl2	1542,08835	1967,42778	1116,74891	0,56761876	-0,81700583	850,6788729	0,02904845
Ap2a1	982,932621	1252,12478	713,740466	0,57002343	-0,81090686	538,3843111	0,0351193
Atp9a	5152,81284	6547,63978	3757,9859	0,57394512	-0,8010153	2789,653882	0,03572765
Aph1a	367,452339	466,033033	268,871645	0,57693688	-0,7935146	197,1613878	0,04697265
Thop1	479,706787	607,813388	351,600187	0,57846733	-0,78969262	256,2132016	0,04253399
Mmp17	899,568925	1135,84792	663,289926	0,58396015	-0,77605817	472,5579975	0,03949969
Spryd3	1472,64822	1857,72542	1087,57103	0,58543153	-0,77242766	770,1543939	0,04490443
Atf6b	477,884724	599,788703	355,980745	0,59351025	-0,75265514	243,8079584	0,04703071
Prrt2	1245,62975	1558,1521	933,107403	0,59885514	-0,73972102	625,0447013	0,04286362
Npy	622,984744	440,761683	805,207806	1,82685528	0,86936235	-364,4461238	0,03205418
Angpt1	208,593358	146,806292	270,380424	1,84174956	0,8810769	-123,5741315	0,04943649
Hnrnpa0	1512,13719	1061,63295	1962,64143	1,84870056	0,88651157	-901,0084791	0,03421167
Slc8a2	132,597519	89,2665704	175,928467	1,9708214	0,97879704	-86,66189643	0,04214033
Fosl2	154,642566	103,423358	205,861775	1,99047662	0,99311392	-102,4384172	0,04903831
Slc17a8	547,595888	365,483833	729,707943	1,99655327	0,99751157	-364,2241101	0,02402521
3110047P20I	133,306586	86,7577906	179,855381	2,07307471	1,05177211	-93,09759069	0,03864048
Plp1	170,657636	110,721942	230,593331	2,08263446	1,05840964	-119,8713896	0,02848706
G530011O06	152,855287	98,6364866	207,074087	2,099366	1,06995371	-108,4376	0,04320019
Antxr1	78,6906126	50,4512664	106,929959	2,11947026	1,08370372	-56,47869237	0,03723475
Map3k6	103,073571	65,9905254	140,156616	2,12388998	1,08670904	-74,16609059	0,0373415
Ubqln2	1748,7269	1116,88134	2380,57246	2,13144617	1,09183262	-1263,691121	0,01063588
2610507I01R	108,935812	69,5580049	148,313618	2,13222933	1,09236262	-78,75561354	0,03403249
Nedd9	92,61843	57,9783603	127,2585	2,19493099	1,13417558	-69,28013942	0,02232248
Sncaip	100,505851	61,8576385	139,154064	2,24958578	1,16965938	-77,29642528	0,02311464
Rpia	45,359313	27,5198342	63,1987919	2,29648157	1,1994252	-35,67895773	0,04943376
Kdm5d	86,4944391	52,1180992	120,870779	2,31917089	1,21360913	-68,75267962	0,00731248
B230120H23	45,0794598	26,8511509	63,3077688	2,35773018	1,23739862	-36,45661792	0,03359127
Ddx3y	941,500992	554,817496	1328,18449	2,39391241	1,25937036	-773,3669908	0,00080068
Uty	151,111228	86,4938668	215,72859	2,49414898	1,31854764	-129,2347228	0,00174375
Pcdh20	65,3737841	36,8715643	93,876004	2,54602716	1,34824781	-57,00443976	0,01249246
Tuba1c	219,309609	119,749532	318,869686	2,66280528	1,41294693	-199,1201539	0,00253804

Tbc1d1	50,6405785	27,1302544	74,1509026	2,73314439	1,45056168	-47,02064817	0,01600824
