# Supplementary material for: SSRIs target prefrontal to raphe circuits during development modulating synaptic connectivity and emotional behavior
Source: Mol Psychiatry. 2018 Oct 2;24(5):726–45. doi: 10.1038/s41380-018-0260-9 (PMC6445781; doi:10.1038/s41380-018-0260-9)
Supplement: Supplementary file 3 — supplementary table 4 [file 41380_2018_260_MOESM3_ESM.pdf]

Current Gene List: new_converted_list
Current Background: Mus musculus
120 DAVID IDs

Options

241 chart records Right-click and select 'Save Target As' to download results Download File

Sublist	Category	Term	Count	%	P-Value	Fisher Exact	Fold Enrichment	log10 P-val
	GOTERM_BP_4	regulation of c	2,90E+01	24,2	0,00056	0,00029	1,9	3,537602
	GOTERM_BP_5	modulation o	8,00E+00	6,7	0,0015	0,00031	4,7	3,50863831
	GOTERM_BP_4	modulation o	8,00E+00	6,7	0,0015	0,00031	4,7	3,50863831
	GOTERM_BP_3	modulation o	8,00E+00	6,7	0,0018	0,00038	4,6	3,4202164
	GOTERM_BP_A	modulation o	8,00E+00	6,7	0,0018	0,00038	4,5	3,4202164
	GOTERM_BP_3	regulation of s	2,80E+01	23,3	0,00091	0,00047	1,9	3,32790214
	GOTERM_BP_3	regulation of c	2,90E+01	24,2	0,00092	0,00048	1,8	3,31875876
	GOTERM_BP_A	regulation of s	28	23,3	0,00095	0,00049	1,9	3,30980392
	GOTERM_BP_A	regulation of c	29	24,2	0,00096	0,00051	1,8	3,29242982
	GOTERM_BP_3	regulation of s	28	23,3	0,001	0,00052	1,9	3,28399666
	GOTERM_CC_3	cell projection	14	11,7	0,0021	0,00075	2,6	3,12493874
	GOTERM_CC_3	cell projection	14	11,7	0,0021	0,00075	2,6	3,12493874
	GOTERM_CC_4	cell projection	14	11,7	0,0022	0,0008	2,6	3,09691001
	GOTERM_MF_	kinase binding	11	9,2	0,0032	0,001	3	3
	GOTERM_CC_1	membrane	70	58,3	0,0016	0,0012	1,3	2,92081875
	GOTERM_CC_A	membrane	70	58,3	0,0016	0,0012	1,3	2,92081875
	GOTERM_BP_5	regulation of a	7	5,8	0,0063	0,0014	4,2	2,85387196
	GOTERM_MF_	kinase binding	11	9,2	0,0043	0,0014	2,9	2,85387196
	GOTERM_CC_A	cell projection	14	11,7	0,0042	0,0017	2,4	2,76955108
	GOTERM_BP_A	regulation of a	7	5,8	0,0072	0,0017	4,1	2,76955108
	GOTERM_BP_4	lipid biosynth	9	7,5	0,0059	0,0017	3,3	2,76955108
	GOTERM_BP_A	lipid biosynth	9	7,5	0,007	0,0021	3,2	2,67778071
	GOTERM_MF_	kinase activity	11	9,2	0,0093	0,0034	2,6	2,46852108
	GOTERM_BP_3	regulation of g	10	8,3	0,011	0,0037	2,7	2,43179828
	GOTERM_BP_A	regulation of g	10	8,3	0,011	0,0038	2,7	2,4202164

GOTERM_BP_:	regulation of g	10	8,3	0,011	0,0038	2,7	2,4202164
GOTERM_MF_	metal ion binc	30	25	0,007	0,0043	1,6	2,36653154
GOTERM_MF_	transferase act	12	10	0,011	0,0045	2,4	2,34678749
GOTERM_MF_	kinase activity	11	9,2	0,012	0,0048	2,5	2,31875876
GOTERM_MF_	transferase act	12	10	0,013	0,0055	2,3	2,25963731
GOTERM_BP_	protein phosp	18	15	0,011	0,0056	1,9	2,25181197
GOTERM_MF_	protein kinase	9	7,5	0,016	0,0057	2,7	2,24412514
GOTERM_BP_!	synaptic trans	8	6,7	0,018	0,0057	2,9	2,24412514
GOTERM_BP_	synaptic trans	8	6,7	0,018	0,0057	2,9	2,24412514
GOTERM_MF_	phosphotrans	10	8,3	0,016	0,0059	2,5	2,22914799
GOTERM_BP_	cellular lipid r	11	9,2	0,015	0,0061	2,4	2,21467016
GOTERM_MF_	cation binding	30	25	0,01	0,0064	1,5	2,19382003
GOTERM_MF_	catalytic activ	43	35,8	0,0095	0,0067	1,4	2,1739252
GOTERM_MF_	catalytic activ	43	35,8	0,0095	0,0067	1,4	2,1739252
GOTERM_BP_	synaptic trans	8	6,7	0,021	0,0068	2,9	2,16749109
GOTERM_BP_:	cellular lipid r	11	9,2	0,018	0,0074	2,3	2,13076828
GOTERM_BP_	cellular lipid r	11	9,2	0,018	0,0075	2,3	2,12493874
GOTERM_MF_	ion binding	41	34,2	0,011	0,0077	1,4	2,11350927
GOTERM_MF_	metal ion binc	30	25	0,012	0,0079	1,5	2,10237291
GOTERM_MF_	phosphotrans	10	8,3	0,02	0,008	2,4	2,09691001
GOTERM_BP_!	axon developr	7	5,8	0,026	0,008	3,1	2,09691001
GOTERM_BP_	response to nu	8	6,7	0,024	0,0081	2,8	2,09151498
GOTERM_MF_	protein kinase	9	7,5	0,023	0,0087	2,5	2,06048075
GOTERM_MF_	ion binding	41	34,2	0,013	0,0088	1,4	2,05551733
GOTERM_MF_	cation binding	30	25	0,014	0,0091	1,5	2,04095861
GOTERM_BP_	axon developr	7	5,8	0,029	0,0093	3	2,03151705
GOTERM_BP_	response to nu	8	6,7	0,027	0,0096	2,7	2,01772877
GOTERM_BP_	cell-cell signal	11	9,2	0,023	0,01	2,2	2
GOTERM_BP_!	phosphorylati	19	15,8	0,018	0,01	1,7	2
GOTERM_CC_:	intracellular n	66	55	0,012	0,01	1,2	2
GOTERM_CC_!	intracellular n	66	55	0,013	0,011	1,2	1,95860731
GOTERM_CC_	intracellular n	66	55	0,014	0,011	1,2	1,95860731
GOTERM_BP_:	cell-cell signal	11	9,2	0,028	0,012	2,2	1,92081875
GOTERM_MF_	protein serine	7	5,8	0,037	0,012	2,8	1,92081875

GOTERM_BP_	cell-cell signal	11	9,2	0,028	0,012	2,2	1,92081875
GOTERM_BP_	response to ex	8	6,7	0,034	0,012	2,6	1,92081875
GOTERM_BP_	response to ex	8	6,7	0,034	0,013	2,6	1,88605665
GOTERM_CC_	endosome	9	7,5	0,033	0,013	2,4	1,88605665
GOTERM_BP_	single-organis	14	11,7	0,027	0,013	1,9	1,88605665
GOTERM_CC_	endosome	9	7,5	0,034	0,014	2,4	1,85387196
GOTERM_BP_	phosphorylati	19	15,8	0,025	0,014	1,7	1,85387196
GOTERM_BP_	single-organis	14	11,7	0,028	0,014	1,9	1,85387196
GOTERM_CC_	cell periphery	35	29,2	0,021	0,014	1,4	1,85387196
GOTERM_CC_	cell periphery	35	29,2	0,021	0,014	1,4	1,85387196
GOTERM_CC_	membrane-bo	71	59,2	0,017	0,015	1,2	1,82390874
GOTERM_CC_	endosome	9	7,5	0,038	0,016	2,3	1,79588002
GOTERM_CC_	cell part	88	73,3	0,018	0,017	1,1	1,76955108
GOTERM_CC_	plasma memb	34	28,3	0,024	0,017	1,4	1,76955108
GOTERM_CC_	plasma memb	34	28,3	0,025	0,017	1,4	1,76955108
GOTERM_BP_	establishment	20	16,7	0,031	0,018	1,6	1,74472749
GOTERM_MF_	protein kinase	8	6,7	0,047	0,019	2,4	1,7212464
GOTERM_BP_	regulation of	6	5	0,057	0,019	2,9	1,7212464
GOTERM_BP_	regulation of	6	5	0,057	0,019	2,9	1,7212464
GOTERM_CC_	plasma memb	34	28,3	0,027	0,019	1,4	1,7212464
GOTERM_BP_	establishment	20	16,7	0,032	0,019	1,6	1,7212464
GOTERM_MF_	transferase ac	20	16,7	0,032	0,019	1,6	1,7212464
GOTERM_CC_	cytoplasmic p	47	39,2	0,025	0,019	1,3	1,7212464
GOTERM_CC_	synapse part	8	6,7	0,05	0,02	2,4	1,69897
GOTERM_BP_	positive regula	14	11,7	0,038	0,02	1,8	1,69897
GOTERM_MF_	transferase ac	20	16,7	0,034	0,02	1,6	1,69897
GOTERM_BP_	regulation of	6	5	0,062	0,021	2,8	1,67778071
GOTERM_BP_	response to ox	13	10,8	0,041	0,021	1,9	1,67778071
GOTERM_BP_	regulation of	6	5	0,063	0,021	2,8	1,67778071
GOTERM_CC_	endosome	9	7,5	0,05	0,021	2,2	1,67778071
GOTERM_CC_	cytoplasmic p	47	39,2	0,028	0,021	1,3	1,67778071
GOTERM_BP_	response to ox	13	10,8	0,042	0,021	1,9	1,67778071
GOTERM_BP_	regulation of	8	6,7	0,054	0,022	2,3	1,65757732
GOTERM_BP_	regulation of	8	6,7	0,055	0,022	2,3	1,65757732

GOTERM_MF_	enzyme bindir	17	14,2	0,04	0,022	1,7	1,65757732
GOTERM_CC_:	synapse	10	8,3	0,051	0,023	2,1	1,63827216
GOTERM_CC_,	synapse	10	8,3	0,051	0,023	2,1	1,63827216
GOTERM_BP_:	positive regul:	14	11,7	0,046	0,025	1,8	1,60205999
GOTERM_BP_:	lipid metaboli	12	10	0,05	0,025	1,9	1,60205999
GOTERM_BP_·	intracellular t	14	11,7	0,047	0,025	1,8	1,60205999
GOTERM_BP_,	positive regul:	14	11,7	0,047	0,025	1,8	1,60205999
GOTERM_BP_,	lipid metaboli	12	10	0,05	0,025	1,9	1,60205999
GOTERM_CC_:	whole membr	15	12,5	0,046	0,025	1,7	1,60205999
GOTERM_BP_:	positive regul:	14	11,7	0,048	0,026	1,8	1,58502665
GOTERM_BP_,	regulation of (	8	6,7	0,062	0,026	2,3	1,58502665
GOTERM_BP_,	regulation of p	13	10,8	0,05	0,026	1,8	1,58502665
GOTERM_BP_:	signaling	43	35,8	0,035	0,026	1,3	1,58502665
GOTERM_BP_,	signaling	43	35,8	0,035	0,026	1,3	1,58502665
GOTERM_BP_,	regulation of p	8	6,7	0,064	0,027	2,2	1,56863624
GOTERM_MF_	protein kinase	8	6,7	0,063	0,027	2,2	1,56863624
GOTERM_CC_!	cytoplasmic p	47	39,2	0,034	0,027	1,2	1,56863624
GOTERM_MF_	purine ribonu	16	13,3	0,047	0,027	1,7	1,56863624
GOTERM_CC_:	Golgi apparatu	6	5	0,076	0,027	2,6	1,56863624
GOTERM_MF_	ribonucleosid	16	13,3	0,048	0,027	1,7	1,56863624
GOTERM_MF_	purine nucleo	16	13,3	0,048	0,027	1,7	1,56863624
GOTERM_MF_	identical prot	13	10,8	0,052	0,027	1,8	1,56863624
GOTERM_MF_	enzyme bindir	17	14,2	0,048	0,028	1,6	1,55284197
GOTERM_CC_·	Golgi apparatu	6	5	0,078	0,028	2,6	1,55284197
GOTERM_MF_	nucleoside bir	16	13,3	0,049	0,028	1,7	1,55284197
GOTERM_BP_·	autophagy	6	5	0,079	0,028	2,6	1,55284197
GOTERM_MF_	protein bindir	54	45	0,035	0,028	1,2	1,55284197
GOTERM_BP_!	regulation of t	9	7,5	0,064	0,029	2,1	1,537602
GOTERM_BP_·	regulation of t	9	7,5	0,064	0,029	2,1	1,537602
GOTERM_BP_!	blood circulat	6	5	0,081	0,029	2,6	1,537602
GOTERM_CC_:	synapse part	8	6,7	0,07	0,03	2,2	1,52287875
GOTERM_CC_,	synapse part	8	6,7	0,07	0,03	2,2	1,52287875
GOTERM_BP_·	circulatory sy:	6	5	0,083	0,03	2,6	1,52287875
GOTERM_CC_!	Golgi apparatu	6	5	0,084	0,031	2,5	1,50863831

GOTERM_BP_	regulation of r	26	21,7	0,046	0,031	1,4	1,50863831
GOTERM_BP_	regulation of p	15	12,5	0,055	0,031	1,7	1,50863831
GOTERM_MF_	nucleotide bir	19	15,8	0,051	0,031	1,6	1,50863831
GOTERM_MF_	nucleoside ph	19	15,8	0,051	0,031	1,6	1,50863831
GOTERM_BP_	intracellular t	14	11,7	0,057	0,031	1,7	1,50863831
GOTERM_MF_	purine ribonu	16	13,3	0,055	0,031	1,6	1,50863831
GOTERM_BP_	cell communi	44	36,7	0,041	0,031	1,3	1,50863831
GOTERM_BP_	regulation of r	26	21,7	0,048	0,032	1,4	1,49485002
GOTERM_BP_	autophagy	6	5	0,087	0,032	2,5	1,49485002
GOTERM_MF_	identical prot	13	10,8	0,061	0,033	1,7	1,48148606
GOTERM_BP_	regulation of r	26	21,7	0,049	0,033	1,4	1,48148606
GOTERM_MF_	purine nucleo	16	13,3	0,057	0,033	1,6	1,48148606
GOTERM_BP_	blood circulat	6	5	0,089	0,033	2,5	1,48148606
GOTERM_BP_	cell communi	44	36,7	0,043	0,033	1,3	1,48148606
GOTERM_MF_	protein bindir	54	45	0,041	0,033	1,2	1,48148606
GOTERM_MF_	ribonucleotid	16	13,3	0,058	0,034	1,6	1,46852108
GOTERM_BP_	circulatory sy:	6	5	0,091	0,034	2,5	1,46852108
GOTERM_BP_	regulation of t	9	7,5	0,074	0,034	2	1,46852108
GOTERM_MF_	nucleoside bir	16	13,3	0,059	0,034	1,6	1,46852108
GOTERM_BP_	regulation of l	8	6,7	0,079	0,035	2,1	1,45593196
GOTERM_BP_	regulation of c	8	6,7	0,084	0,037	2,1	1,43179828
GOTERM_BP_	regulation of c	8	6,7	0,084	0,037	2,1	1,43179828
GOTERM_CC_	Golgi apparatu	6	5	0,099	0,038	2,4	1,4202164
GOTERM_BP_	positive regula	14	11,7	0,068	0,038	1,7	1,4202164
GOTERM_MF_	ribonucleosid	16	13,3	0,065	0,038	1,6	1,4202164
GOTERM_BP_	positive regula	14	11,7	0,068	0,038	1,7	1,4202164
GOTERM_MF_	purine nucleo	16	13,3	0,065	0,039	1,6	1,40893539
GOTERM_BP_	regulation of p	15	12,5	0,068	0,039	1,6	1,40893539
GOTERM_MF_	anion binding	21	17,5	0,061	0,039	1,5	1,40893539
GOTERM_MF_	nucleotide bir	19	15,8	0,063	0,039	1,5	1,40893539
GOTERM_MF_	nucleoside ph	19	15,8	0,063	0,039	1,5	1,40893539
GOTERM_BP_	single organisı	42	35	0,051	0,039	1,3	1,40893539
GOTERM_BP_	regulation of l	8	6,7	0,089	0,04	2,1	1,39794001
GOTERM_BP_	regulation of s	19	15,8	0,065	0,04	1,5	1,39794001

GOTERM_BP_	regulation of	19	15,8	0,065	0,041	1,5	1,38721614
GOTERM_BP_	negative regul	11	9,2	0,079	0,041	1,8	1,38721614
GOTERM_BP_	negative regul	11	9,2	0,079	0,041	1,8	1,38721614
GOTERM_BP_	single organis	42	35	0,053	0,041	1,3	1,38721614
GOTERM_BP_	negative regul	7	5,8	0,097	0,041	2,2	1,38721614
GOTERM_MF_	ribonucleotid	16	13,3	0,069	0,041	1,6	1,38721614
GOTERM_CC_	Golgi apparat	12	10	0,077	0,042	1,7	1,37675071
GOTERM_BP_	phosphate-co	22	18,3	0,064	0,042	1,4	1,37675071
GOTERM_BP_	regulation of	8	6,7	0,093	0,042	2	1,37675071
GOTERM_BP_	regulation of	8	6,7	0,094	0,043	2	1,36653154
GOTERM_BP_	regulation of	13	10,8	0,078	0,043	1,7	1,36653154
GOTERM_CC_	Golgi apparat	12	10	0,081	0,044	1,7	1,35654732
GOTERM_MF_	purine ribonu	16	13,3	0,074	0,044	1,6	1,35654732
GOTERM_BP_	localization	38	31,7	0,06	0,045	1,3	1,34678749
GOTERM_BP_	localization	38	31,7	0,06	0,045	1,3	1,34678749
GOTERM_BP_	protein metab	35	29,2	0,061	0,045	1,3	1,34678749
GOTERM_MF_	carbohydrate	18	15	0,073	0,045	1,5	1,34678749
GOTERM_MF_	purine ribonu	16	13,3	0,075	0,045	1,6	1,34678749
GOTERM_MF_	nucleotide bi	19	15,8	0,072	0,046	1,5	1,33724217
GOTERM_BP_	neuron differe	12	10	0,085	0,046	1,7	1,33724217
GOTERM_MF_	purine nucleo	16	13,3	0,077	0,046	1,6	1,33724217
GOTERM_BP_	positive regul	14	11,7	0,081	0,047	1,6	1,32790214
GOTERM_BP_	regulation of	9	7,5	0,096	0,047	1,9	1,32790214
GOTERM_BP_	regulation of	9	7,5	0,096	0,047	1,9	1,32790214
GOTERM_CC_	whole membr	15	12,5	0,08	0,047	1,6	1,32790214
GOTERM_BP_	positive regul	14	11,7	0,082	0,047	1,6	1,32790214
GOTERM_BP_	growth	10	8,3	0,093	0,047	1,8	1,32790214
GOTERM_BP_	growth	10	8,3	0,093	0,047	1,8	1,32790214
GOTERM_MF_	ribonucleotid	16	13,3	0,078	0,047	1,6	1,32790214
GOTERM_BP_	establishment	15	12,5	0,081	0,048	1,6	1,31875876
GOTERM_CC_	cell periphery	35	29,2	0,065	0,048	1,3	1,31875876
GOTERM_BP_	negative regul	11	9,2	0,091	0,048	1,8	1,31875876
GOTERM_MF_	carbohydrate	18	15	0,077	0,048	1,5	1,31875876
GOTERM_MF_	purine ribonu	15	12,5	0,082	0,048	1,6	1,31875876

GOTERM_BP_	negative regul	11	9,2	0,092	0,049	1,8	1,30980392
GOTERM_CC_	plasma memb	9	7,5	0,1	0,049	1,9	1,30980392
GOTERM_MF_	anion binding	21	17,5	0,075	0,049	1,4	1,30980392
GOTERM_CC_	Golgi apparatu	12	10	0,09	0,05	1,7	1,30103
GOTERM_BP_	regulation of p	14	11,7	0,086	0,05	1,6	1,30103
GOTERM_BP_	neuron develo	10	8,3	0,098	0,051	1,8	1,29242982
GOTERM_BP_	neuron develo	10	8,3	0,098	0,051	1,8	1,29242982
GOTERM_BP_	protein transp	14	11,7	0,088	0,051	1,6	1,29242982
GOTERM_BP_	protein transp	14	11,7	0,088	0,051	1,6	1,29242982
GOTERM_BP_	regulation of s	19	15,8	0,08	0,051	1,5	1,29242982
GOTERM_BP_	regulation of p	14	11,7	0,088	0,051	1,6	1,29242982
GOTERM_BP_	regulation of p	14	11,7	0,088	0,052	1,6	1,28399666
GOTERM_MF_	adenyl ribonu	13	10,8	0,092	0,052	1,6	1,28399666
GOTERM_BP_	regulation of s	19	15,8	0,082	0,052	1,5	1,28399666
GOTERM_MF_	purine ribonu	16	13,3	0,086	0,053	1,5	1,27572413
GOTERM_CC_	plasma memb	34	28,3	0,073	0,054	1,3	1,26760624
GOTERM_MF_	adenyl nucleo	13	10,8	0,095	0,054	1,6	1,26760624
GOTERM_MF_	purine nucleo	16	13,3	0,089	0,055	1,5	1,25963731
GOTERM_BP_	neuron differe	12	10	0,099	0,056	1,7	1,25181197
GOTERM_BP_	phosphate-co	22	18,3	0,084	0,056	1,4	1,25181197
GOTERM_MF_	ribonucleotid	16	13,3	0,091	0,056	1,5	1,25181197
GOTERM_BP_	regulation of b	24	20	0,083	0,057	1,4	1,24412514
GOTERM_BP_	regulation of c	18	15	0,09	0,057	1,5	1,24412514
GOTERM_BP_	regulation of c	18	15	0,09	0,057	1,5	1,24412514
GOTERM_CC_	intracellular	77	64,2	0,064	0,058	1,1	1,23657201
GOTERM_BP_	cellular locali:	21	17,5	0,087	0,058	1,4	1,23657201
GOTERM_BP_	establishment	15	12,5	0,096	0,058	1,5	1,23657201
GOTERM_BP_	regulation of b	24	20	0,085	0,059	1,4	1,22914799
GOTERM_CC_	intracellular	77	64,2	0,066	0,059	1,1	1,22914799
GOTERM_BP_	establishment	15	12,5	0,097	0,059	1,5	1,22914799
GOTERM_BP_	cellular locali:	21	17,5	0,089	0,059	1,4	1,22914799
GOTERM_BP_	intracellular s	18	15	0,094	0,061	1,5	1,21467016
GOTERM_BP_	intracellular s	18	15	0,095	0,061	1,5	1,21467016
GOTERM_BP_	protein modif	24	20	0,09	0,063	1,4	1,20065945

GOTERM_BP_!	cellular protei	24	20	0,09	0,063	1,4	1,20065945
GOTERM_BP_	protein modif	24	20	0,091	0,063	1,4	1,20065945
GOTERM_CC_:	cytoplasm	62	51,7	0,074	0,063	1,1	1,20065945
GOTERM_BP_:	phosphorus m	22	18,3	0,093	0,063	1,4	1,20065945
GOTERM_BP_	phosphorus m	22	18,3	0,095	0,065	1,4	1,18708664
GOTERM_BP_:	protein metab	35	29,2	0,087	0,066	1,3	1,18045606
GOTERM_BP_	protein metab	35	29,2	0,089	0,068	1,3	1,16749109
GOTERM_CC_	cytoplasm	62	51,7	0,084	0,072	1,1	1,1426675
GOTERM_BP_!	cellular protei	30	25	0,099	0,073	1,3	1,13667714
GOTERM_BP_	cellular protei	30	25	0,1	0,074	1,3	1,13076828
GOTERM_CC_:	intracellular c	67	55,8	0,092	0,081	1,1	1,09151498
GOTERM_CC_:	intracellular c	67	55,8	0,094	0,082	1,1	1,08618615

11 from your list are not in the output,
